# Part I. Spirit of English Periodical Literature

**Published:** 1833-07-01

**Authors:** 


### Periscope;

OR,

### CIRCUMSPECTIVE REVIEW.

"Ore trahit quodcunque potest, atque addit acervo."

I.

### Spirit of English Periodical Literature, &c.

### I. Dr. Graves on the Treatment of Various Diseases.

Dr. G. is engaged, and very profitably too, in publishing a sort of catalogue raisonné of his experience. It is a refreshing thing to turn occasionally from systems and generalizations to the raw material, and add some facts to our own stock in trade. The first subject to which Dr. Graves adverts is that of

#### Headaches in Young Women.

He observes that when connected with an obviously plethoric state of body, the treatment is sufficiently well understood—early hours, spare diet, active exercise, rather powerful purgatives. When there is much determination of blood to the head, leeches may be applied behind to the ears, or to the feet, and the bleeding in the *latter instance should be kept up* by the pediluvium. Dr. G. speaks very highly in favour of the latter method, and illustrates its good effects, by relating the case of an old gentleman, who was subject to attacks of violent palpitations, accompanied by the feeling of approaching dissolution. Dr. G. saw him in a very severe paroxysm, in which the ordinary means had failed. A pediluvium as hot as it could possibly be borne afforded speedy and decisive relief. In the habitual headaches of robust and plethoric young women, it is sometimes necessary to have recourse to general blood-letting when the paroxysm is violent. A young lady had been subject for years to attacks of most distressing severity, which baffled all internal remedies and external applications. At length Dr. Stokes bled her ad deliquium, during a violent paroxysm, with immediate, and what is more, with permanent relief, for she has had no subsequent attack.

Dr. Graves makes some remarks on the treatment of amenorrhœa, which are practically so judicious that we feel anxious to impress them on the minds of our readers.

"The periodicity of this function can still be traced, even in cases where suppression has continued for a great length of time by means of the menstrual *molimina,* which occur at stated intervals; in endeavouring to bring on the discharge, therefore, we must be guided as to the time the attempt should be made by an observance of the period at which these *molimina* occur; for a few days before that time, our efforts to produce a determination of blood to the uterus may be judiciously employed, and if they fail, the attempt should be abandoned, until a few days before the next menstrual period: of course I speak not here of the general constitutional treatment, for this must be constantly persevered in, one of the chief means of bringing back this evacuation being the restoration of the health to the natural standard; in some this is to be effected by tonic, and in others by an opposite mode of general treatment.

But of this it is quite unnecessary to speak, as all practitioners are acquainted with the essential difference between the general modes of management required according to the constitution and habits of the patient. What I wish to impress on the minds of the junior members of the profession is, that all those remedies which actually determine to the uterus or its neighbourhood, as pediluvia, stuping of the genitals, leeches to the inside of the thighs near the labia, aloes and other stimulating purgatives, &c. &c. should be only used at the times already spoken of. To use them at any other period, either after the molimina have disappeared, or during the intervals between them, tends in most cases still further to derange nature, by determining to the uterus at an unseasonable time, when there is no natural tendency to that organ; under such circumstances the very same means will frequently fail and prove injurious, which, applied so as to coincide with the time of the natural effort, would have been successful. To illustrate these principles by an example: we are consulted in the case of a young woman, affected with various hysterical symptoms for several months, and during that period more than usually subject to headach, languor, loss of spirits, diminution of appetite, and irregularity, usually constipation of bowels; she is pale, and complains of various pains and uneasy sensations, and has not menstruated since the accession of these symptoms; here it is evident that the constitutional treatment must be strengthening and tonic; the practitioner will therefore recommend regular hours, much gestation in the open air, a nutritious diet, tepid, and afterwards cold shower baths; he will regulate the bowels and afterwards prescribe a course of tonic medicines, chalybeates, preparations of bark, strychnine, &c. &c.; he will likewise inquire carefully when the last period happened, and when and how often since that occurrence menstrual molimina were observed. He thus ascertains when they should again recur, and contents himself with enforcing the constitutional treatment, until about six days before the calculated time. Then he lays aside the other medicines, and has recourse to those means which determine to the uterus. Two leeches are applied to the inside of the thigh near the labium, every second night, until they have been three times applied. The bleeding is encouraged by stuping. On the intermediate days the bowels must be actively moved by aloetic pills, and for three nights before and after the day of the molimina,* hot pediluvia, rendered stimulating by mustard seed, may be used; during the same time also frictions with stimulating liniments should be applied to the feet and legs every morning, and spirits of turpentine or tincture of cantharides may be exhibited internally, while the necessity of more active exercise is inculcated. The intention of the leeching is to produce a tendency of blood to the part, which tendency is increased by each repetition of the application, and is still further augmented by these applications being made only about the time that the menstrual discharge should have taken place. *If these means fail, they must for the moment be laid aside, and the constitutional treatment must be again resumed until the same number of days before the next period, when the list of remedies above spoken of must be again tried,* and in few cases indeed shall we find them to fail."

We can add our testimony to that of Dr. Graves, respecting the superior efficacy of this over the routine administration of emmenagogues, tonics, and so forth, without regard to the periods that nature has assigned, and the indications of them that she offers. Dr. Graves omits mentioning the hip-bath, which we have found preferable to the pediluvium. Dr. G. observes that two leeches are a fit number for weakly individuals, but in the plethoric, four or six may be used at a time with advantage.

---

* "By *molimina* are meant pains in the loins, thighs, and hypogastric region, flushings, colicky pains of the abdomen, increase of headach, and a general feeling of malaise, which are familiarly known among females as indicating a *constitutional effort*."

Dr. Graves observes that in some young women the tendency to headaches exists without any menstrual derangement, but may be caused by leucorrhœa. When this is the case Dr. G. counsels avoidance of the pediluvia, and the use of the nitrate of silver injection, and he adds that the removal of the leucorrhœa is the first step in the cure, as it causes a series of most distressing symptoms. We must confess that our experience on this head does not altogether tally with that of Dr. Graves. In young women leucorrhœa is usually merely a symptom, and it is not this discharge, but the state of the general habit on which it depends that is the fons et origo mali. It is true that by removing the leucorrhœa we effect much good, but in fact we do not cure the leucorrhœa until we have altered and ameliorated the general health.

Dr. Graves next speaks of headache in young persons of a delicate excitable temperament without any menstrual or leucorrhœal complication.

Such persons are said to be extremely nervous, and are subject to every variety of hysterical seizure, all, however, marked by the violence of the accompanying headache. In some the pain is accompanied with flushed countenance, in others the external signs of cerebral congestion are less evident, but in all, any stimulus, such as wine, aggravates the malady. These patients are often kept awake night after night —they bear active purgation very badly, and loss of blood, whether general or local, as well as blisters, and means of this description, although they may procure a temporary alleviation, do ultimate injury. Dr. Graves alludes to a fact illustrative of this circumstance. A lady had been liable every third or fourth month for the last twenty-five years to a violent fit of epilepsy ; about a year ago a young practitioner imprudently bled her, and she has since been subject to an attack every third or fourth week.

Dr. G. observes that we should recollect that the natural tendency of this complaint, when not interfered with by art, is by no means dangerous, although it is, of course, an object to relieve the patient of so distressing a symptom as speedily as possible. The means which Dr. G. employs are these : first, moderately cold applications to the forehead ; secondly, attention to the bowels by means of fœtid and terebinthinate enemata, at least once a day ; thirdly, attention to the state of the bladder, lest water should accumulate, as it frequently does in that organ ; fourthly, extensive, diligent, and frequently repeated dry cupping of the integuments in the vicinity of the head ; fifthly, the internal exhibition of spirit of turpentine in considerable doses ; sixthly, the repeated use of stimulating liniments to the abdomen and the lower extremities ; and, lastly, when the fit has subsided, or other remedies have failed, the *nitrate of silver*, in considerable doses.

"The utility of both nitrate of silver and spirit of turpentine in such cases, was suggested to me by the good effects these medicines are found to produce in epilepsy, particularly when it occurs in persons of a nervous and delicate habit, and since I have employed them in hysterical determination to the head, I have been able to overcome these and similar affections, with much greater facility than formerly : of these, as has been already observed, the spirit of turpentine is best suited to the violent stages of the disorder, and may be given in doses of one or two drachms, to be repeated according to its effects. The best vehicle is cold water ; some will bear and derive advantage from two or three doses of this medicine in the day, experiencing from its use a diminution of headache, and removal of flatulence, together with a moderate action of the bowels and kidneys. In some cases, as occurs also occasionally in the treatment of epilepsy, by this medicine, it cannot be persevered in, in consequence of the violent dysuria and hæmaturia it occasions ; slighter degrees of these affections should not, however, prevent our continuing it. When the paroxysm has abated, or when the spirit of turpentine has failed, the greatest benefit may be derived from the nitrate of silver continued for five or six days at a time, in doses of half a grain four times, or even six times a day. When the bowels are constipated there is no better combination than nitrate of silver with minute doses of compound colocynth pill, a formula, I believe first recommended in dyspepsia by Doctor James Johnson, of London, and which I have found invaluable, not merely in the headachs of hysterical young women, but in those of men, particularly the habitual stomach headach, to which delicate and literary men are so subject."

Dr. Graves speaks very highly of dry cupping. In another part of this Journal will be found a brief notice of a valuable paper on this subject in the Lancet, from the pen of Mr. Roberton. Dr. G. recommends that many cups, of tolerable size should be applied at once to the nape of the neck, between the shoulders and below the clavicles, whilst one or two small ones may be applied near the ears. The suction should be powerful, and should be sufficient to fix the cup for at least ten or fifteen minutes. Dr. G. recommends dry cupping in hysterical headache, coma, and delirium, as well as previously to the occurrence of epileptic paroxysms. He mentions cases in point. With reference to hysterical delirium we should state, that our author describes it, as characterised by great nervous excitement, sleeplessness, talkativeness, and delusions, such as supposing persons to be present who are not so, accompanied with a frequent wish to get out of bed in some, while others hide themselves under the clothes, when a stranger approaches.

In such cases Dr. Graves has known the most disastrous consequences result from the depleting system being solely relied on; he recommends the dry cupping as likely to prove a most valuable auxiliary to well-directed internal treatment. Mr. Barker communicated a curious case to our author. A lady of rank was occasionally attacked by violent determination to the head, and each of these paroxysms was sure to induce before it ended a great propensity to suicide, which on one occasion she nearly succeeded in effecting. This strange affection was removed, or rather prevented, by the application of dry cupping as soon as the premonitory symptoms of the paroxysm made their appearance.

### EXHIBITION OF OPIUM IN THE FORM OF ENEMA.

Dr. Graves relates two cases illustrative of the efficacy of opium when given as an injection. Singularly enough, the subjects of both cases are medical men. The first was a gentleman worn down with the effects of mercury. He had chronic arthritic swellings, slight rupia, pains, extreme debility, and an utter want of sleep. For two years he had never slept at night without the assistance of an opiate, and he had often taken two ounces of Battley's solution in the day. Very large doses of opium acted on his bowels as an aperient, and it never checked the secretions or induced pyrexia. The treatment pursued by Dr. Graves consisted in the administration of two drachms of the black drop every night—three drops of Fowler's solution three times daily — a pint of sarsaparilla broth—a starch enema, with one scruple of black drop three times daily—a nutritious but mild diet—some wine at dinner. This treatment was attended with the happiest effects.

The other case was one of neuralgia. The gentleman has frequently been compelled to take during the paroxysms 100 grains of opium, which produced disturbance of the secretions, destroyed the appetite, and had sensibly impaired the memory and mental powers. Half a drachm of laudanum used in the form of injection twice or three times daily effectually alleviates his suffering and produces none of the bad consequences alluded to.

We must confess that we have seen opium given by injections frequently fail in producing the same amount of relief with its exhibition by the mouth. Since the publication of M. Dupuytren's opinions on the employment of opiate enemata in delirium traumaticum, it has been the fashion to resort to them. In some instances we have certainly observed great benefit from their administration, but in others, as we have already remarked, we have seen them fail. It is always right to make trial of them.

### Dysphagia.

We give the following case of hysteria in our author's words; it is equally brief and instructive.

"On the 1st of last September I was called to see a young lady, who was represented to be in a state of imminent danger. On entering the room I found her sitting up in bed, surrounded by several female friends, all in the greatest alarm. Her face was pale, and her countenance indicated a good deal of anxiety. She held in her right hand a cup containing water, which she applied to her lips about every five seconds, and sipped an extremely small portion of the water, which she immediately swallowed with a considerable effort of deglutition, although the quantity was so trifling; she said that she should be immediately choked if she discontinued this perpetual sipping, and she referred to an intolerable uneasiness at the root of her tongue and in her throat, threatening immediate suffocation the moment she ceased to employ herself in swallowing; and so urgent was the feeling that impelled her to this act, that the moment an attempt was made to take the cup out of her hand, she began to scream with agony, was agitated with convulsions, and to all appearances seemed in the last agony. This scene had lasted for several hours without interruption, and the appearance of the principal actress was rendered still more tragical by a black mass of leeches around her throat, and the blood from their bites trickling down her neck. On examining her more closely I found that there was no obstruction whatsoever to the passage of air through the larynx, and that she could make a full inspiration, without any wheezing or noise in her chest; there was no swelling or redness observable at the root of the tongue, or in the fauces. As the young lady was of an extremely delicate and nervous habit, being very sedentary and subject to frequent attacks of common hysteria, I immediately conjectured that her present symptoms were the result of an hysterical affection, and accordingly I removed the leeches, stopped the bleeding as soon as possible, and gave her draughts consisting of camphor, aromatic spirit of ammonia, and black drop, under the influence of which the nervous irritation soon subsided, and she fell asleep."

Dr. Graves makes mention of two other cases of, apparently, nervous dysphagia. A nervous young clergyman consulted him last year on account of debility and dyspepsia, accompanied with a painful and convulsive struggle, as he expressed it, which sometimes took place between the morsel he had swallowed, just before it entered the stomach, and a something that seemed to resist its further passage downwards. This lasted for a few seconds only, but was very distressing to himself and the spectators, and made him shun society. The other case is that of an excellent anatomist, in whom these sudden attacks of temporary dysphagia have become so habitual that he never ventures to eat unless a glass of water be within his reach; in him, the stoppage of the descent of the food is attended with an urgent sense of suffocation.

"In fever I have witnessed several times a very peculiar species of dysphagia, evidently occasioned by flatulent distension of the stomach to such an extent that the lower portion of the œsophagus partook of this condition; at least, I conjecture so, for during the struggle of the dysphagic paroxysm, a gurgling noise was heard, as if the bit of food was met by a portion of air contained in the lower part of the œsophagus; my friend, Doctor Autenrieth, of Tubingon, has particularly remarked this symptom, or at least something like it, in what he calls the abdominal typhus fever of young people; for he says, if the patient takes any drink a peculiar gurgling noise is heard as if the fluid was poured into a lifeless bag. Now, in precisely such a case, Mr. Rumly and I saw a young lady affected, in addition to this noise, with so great spasmodic dysphagia, probably from the entrance of wind into the lower end of the œsophagus, that she altogether refused to drink. This phenomenon gradually disappeared, and the lady ultimately recovered; but it deserves to be remarked, that in general this symptom and the gurgling noise, described by Dr. Autenrieth, are very bad omens in fever."

The remaining part of Dr. Graves' paper is not possessed of any peculiar interest, and we therefore pass it over. We would make one observation before we conclude. In the earlier days of physic, when morbid anatomy was uncultivated, and when, consequently, medicine had not the degree of exactness which it now possesses, men were thrown solely on the observation of facts, and a vast body of empiric experience was accumulated. We say empiric, because the nature of disease was unknown, and the observation consisted only in the statement of the results of the application of certain remedies to certain symptoms. Much practical truth was, no doubt, obtained; but, as many symptoms constitute only the common language of certain stages of diseases, totally differing in their characters and seat, it followed that much of the experience was fallacious, and men were ignorant why a drug succeeded in one case and utterly failed in another, which, to their eyes, appeared of a similar description. Hence all the vagueness, and much of the opprobrium, of physic.

When morbid anatomy was first ex-explored, and its vast mines of real and solid information opened, men were dazzled by the glare of the wealth around them, and thought that it would suffice for all their necessities and wishes; in other words, they imagined that, to become good practitioners, it was merely necessary to know the real nature of disease, and the structural changes that accompany and occasion symptoms. The example of France is alone sufficient to display the fallacy of this expectation.

The truth is, that both means are necessary to constitute the knowledge available in practice. We should know the seat and the structural nature of disease, or we sink into empirics, and exactness is lost; we should know the effects of remedies on symptoms, as well as on structural lesions, or we become mere barometers of vital changes diagnosticating, prognosticating, doing every thing but cure.

At the present day, these two roads to knowledge may, happily, be joined. Men conversant in the exact truths of morbid anatomy may set themselves to observe the effects of medicines, and we need not say how incomparably superior the record of their experience is, to that empiric jumble of facts and fancies that has descended to us from our forefathers. We now appreciate the effects of remedies with some measure of certainty—we see how far organic lesions are amenable to treatment, and what medicines or means relieve particular symptoms, or sets of symptoms, not dependent on such lesions. In short, we have now a rational and scientific series of experiments, in clinical observation. It is on this account that we have noticed Dr. Graves' paper so fully, and we trust that it will not be the last of a similar description.—*Dublin Journal, No.* VIII.

---

### II. The Whites and the Blacks.

We are not about to discuss the question of emancipation; that is somewhat too political and too polemic. But our attention has been drawn to some remarks on the subject of negro inferiority in our valuable contemporary, the Phrenological Journal for March, 1833.

Many of our readers are perhaps aware that a society has been formed in America for establishing a colony for free negroes, on the Western Coast of Africa, and that this colony has been termed Liberia. It appears that the whole population of the United States is about 13,000,000, and that of these upwards of 2,000,000 are blacks. Of the 2,000,000 blacks about 330,000 are wholly or partially free. Both the slaves and the free blacks are increasing with great and alarming rapidity. It seems too that the free blacks are, generally speaking, a destitute, depraved, and wretched class. Such are the broad facts, and the question arises—in what way can we best remedy this crying moral and political evil? By emancipation at home, or by deportation to their native continent and native land?

We do not propose to enter on political considerations, but as the question admits of being viewed in a physical or mental light, we shall take the liberty of glancing at it. We must say that we feel disposed to agree with our contemporary in most of the following sentiments and conclusions :—

" Even Mr. Stuart will grant to us, that the actual existence of some millions of blacks in the same community with the whites of the United States, is in itself an enormous political and moral evil. That the black population is, *de facto*, an inferior caste, which, with many individual exceptions, no doubt, is generally degraded, uneducated, and in many instances vicious and depraved; and if it be a scourge to America, the punishment is the natural result of a daring violation by man of a marked appointment of God,—a just retribution for the avarice, rapacity, and cruelty that for ages outraged nature, by tearing the African from the region and the climate for which his Creator had fitted his physical constitution, and mingling him with a race with which incorporation was not designed, if a strong natural repugnance to it is to be received as proof of the Divine intention.

It is wild fanaticism to call this repugnance unchristian, and to denounce a doubt of the power of religion to overcome it as infidelity;—*because* God made all men of one flesh, and Christianity bids us open wide the arms of brotherly love, and take all our brethren of mankind to our bosom. It is a stupid perversion of this religious precept to maintain, that the fulfilment of this duty precludes all change of the Negro's place of residence, and that the American does not in effect hold out to him the arms of brotherly love, by placing him in independence, comparative elevation, and abundance, in another country, instead of degradation and destitution where he is. God made all men of one flesh, but he did not design them all to live in one country, and, however various and unsuitable their aspect and nature, to mix and incorporate. If we look at that well marked and vast peninsula called Africa, we find that equally marked race the Negro, with slight modifications, forming its native population throughout all its regions. We find the temperature of his blood, the chemical action of his skin, the very texture of his wool-like hair, all fitting him for the vertical sun of Africa; and if every surviving African of the present day who is living in degradation and destitution in other lands for which he was never intended, were actually restored to the peculiar land of his peculiar race, in independance and comfort, would even Mr. Stuart venture to affirm that Christianity had been lost sight of by all who had in any way contributed to such a consummation? It matters not to brotherly love on which side of the Atlantic the Negro is made enlightened, virtuous and happy, if he is actually so far blessed; but it does matter on which side of the ocean you place him, when there is only one where he will be as happy and respectable as benevolence would wish to see him, and certainly there a rightly applied morality and religion would sanction his being placed. The incurable evil of the present relation of the whites and the blacks in America is, that incorporation is almost morally impossible. The whites are too numerous in both the sexes, to be driven to intermarriage with the Negroes. Mulattoes are a West Indian, greatly more than an American phenomenon. The distinction in the United States is white or black, with little of the intervening shades of colour. The races do not and will not incorporate. Try the loudest advocate for the 'vincibility' of this prejudice, as it is most unphilosophically called, with this touchstone,—' marry the Negresses to your sons, and give your daughters to Negroes,'—and we shall have a different answer from Nature than we receive from a misplaced religious profession."

We are by no means advocates for negro slavery; on the contrary we abhor it, and the present state of the slaves and the slave-holders is, as our contemporary has justly remarked, an eternal lesson to man, that signal punishment must ultimately follow injustice. But we question whether the white man and the black can co-exist as equals in the same land, and if they do not become incorporated, it follows from the nature of things that, sooner or later, a deadly struggle must ensue, a struggle not merely for mastery, but for existence. Many well-meaning persons entertain a different opinion, and they ground it on the belief, that with wise institutions and a good education the black will display an equal or nearly equal degree of mental attainments with the European; in fact, they believe that the white and the negro have originally the same origin, and consequently have originally the same mental capacity. Of their origin we shall say nothing, but we cannot persuade ourselves that the mind of the European and the African are equal, or can, or will, by the utmost stretch of human ingenuity, be ever made so. The difference of conformation between their skulls is so palpable, that all who run may read it. This is a physical difference, and all admit that whatever the mind may be, the physical brain constitutes its organ, and measures its manifestations. We do not know how this grand physical and mental distinction can be done away with, but by intermarriage, by a blending of the races. Is this likely to occur? Many are willing to give the negro the privileges of the man, but few are in-inclined to receive him as the brother or the son.

History confirms what physical conformation suggests. The negro has never been the dominant but always the depressed race. If, of two wrestlers, one is always down, we may feel assured of his being the weaker.

The belief in negro inferiority constitutes no argument for slavery, unless weakness be a reason for the abuse of strength. But it does appear to constitute a valid argument against an attempt at equalization, for what God has made unlike, man will vainly attempt to assimilate. The experiment must end not only in failure, but in misery and ruin. If it were a question between two people, the conquerers and the conquered, incorporation would, under good institutions, ensue. Such has been the case throughout the greater part of Europe. But when strong religious prejudices or national feelings interfere, incorporation does not take place, and a bloody struggle is the final result. Such is the case with the Greeks and the Turks, such possibly may hereafter be the case in India. We have already said that incorporation of the American and African, or of the Anglo-West Indian and African is improbable in the highest degree. The numbers of the two races must necessarily increase till diminished means of subsistence check it. A contest must then come, and such a contest!

Let the African depart to his native continent, give him freedom, civilization, and then admit him into the family of man as an associate. But we doubt the wisdom of the attempt to amalgamate the white and the black races, and we ground this doubt on their physical and mental differences.

---

### III. DEVELOPMENT OF THE HEAD OF DR. SPURZHEIM.

It has been the fortune of Dr. Spurzheim to see many friends, and still more ardent disciples defend himself and disseminate his doctrines, and the latter have taken deep root and gained extensive credit during his life-time. Whatever may be the fate of the details of the science, whether the localization of the organs be correct, or whether an exact localization be attainable, matters little in comparison with the triumphant establishment of its great principle, the plurality of organs. We say triumphant establishment, for the arguments of those opposed to it have utterly failed to check its progress and a blow has been struck at the doctrine of the indivisibility of the mind, which it never can recover. But it is not our object at present to enter upon any phrenological discussion; we wish only to lay before our readers the phrenological admeasurement of the head of the late Dr. Spurzheim.

DEVELOPMENT.*

1. Amativeness, full or ra. large.. 15
2. Philoprogenitiveness, large.... 18
3. Concentrativeness, ra. small .. 8
4. Adhesiveness, rather large.... 16
5. Combativeness, rather full.... 12
6. Destructiveness, very large .. 20
7. Secretiveness, large.......... 18
8. Acquisitiveness, rather large .. 16
9. Constructiveness, ditto ...... 16
10. Self-Esteem, large .......... 18
11. Love of Approbation, ditto, or very large ................ 19
12. Cautiousness, rather large, or large .................... 17
13. Benevolence, very large ...... 20
14. Veneration, ditto............ 20
15. Firmness, ditto............ 20
16. Conscientiousness, rather large, or large .................. 17
17. Hope, rather full, or full .... 13
18. Wonder, full, or rather large.. 15
19. Ideality, rather large ........ 16
20. Wit, rather large, or large.... 17
21. Imitation, rather large ...... 16
22. Individuality, large.......... 18
23. Form, rather large, or large .. 17
24. Size, large ................ 18
25. Weight, full................ 14
26. Colouring, rather full, or full.. 13
27. Locality, large.............. 18
28. Number, rather full, or full.... 13
29. Order, rather large.......... 16
30. Eventuality, full ............ 14
31. Time, large ................ 18
32. Tune, large ................ 18
33. Language, ra. large, or large.. 17
34. Comparison, very large ...... 20
35. Causality, very large ........ 20

MEASUREMENTS.

| | Inches. |
|---|---|
| From Occipital Spine to Individuality | $7\frac{7}{8}$ |
| Concentrativeness to Comparison | $7\frac{2}{8}$ |
| Ear to Occipital Spine ........ | $4\frac{4}{8}$ |
| —— Individuality .......... | $5\frac{2}{8}$ |
| —— Firmness .............. | $6\frac{1}{2}$ |
| —— Benevolence............ | 6 |
| Destructiveness to Destructiveness | $6\frac{6}{8}$ |
| Secretiveness to Secretiveness .. | $6\frac{4}{8}$ |
| Cautiousness to Cautiousness .. | $5\frac{7}{8}$ |
| Ideality to Ideality ............ | $5\frac{6}{8}$ |
| Constructiveness to Construct... | $5\frac{4}{8}$ |

We need scarcely inform our readers that this excellent man and able philosopher died in America, while engaged in delivering a course of lectures at Boston. His death took place on the 10th November, 1832, after an illness of about three weeks, induced and fatally kept up by his exertions. The symptoms were those of continued fever, and, unfortunately, he refused all active treatment, and displayed, as too many of high intellectual attainments do, that species of irritability, which often sets medicine and nature also at defiance. On examining his body, there were merely some traces of increased vascularity discovered in the arachnoid and pia mater, with adhesion of the colon to the peritoneum in the right iliac fossa.

There is a brief, but very interesting account of his life in the Phrenological Journal. Mr. Holm is preparing a more extensive biography.—*Phrenological Journal, No.* XXXV.

* "The numbers on the right indicate the size of the organs according to the scale adopted by the Phrenological Society, and described in Combe's System, p. 95."

---

### IV. The Pathology of Purpura.

Mr. Gardner, of Glasgow, has written a paper on this recondite subject, in the Glasgow Medical Journal, for April of the present year. After discussing all the theories that have hitherto been offered, he proposes his own views, which we fear are as open to criticism as those of his predecessors.

Dr. Hannay, Professor of Physic in the Andersonian University, has asserted that the disease is always produced by chronic inflammation of the veins, verging more or less to an acute form; and he grounds his opinion on the fact that in three successive dissections of patients who died of purpura, the veins were found in a high state of disorganization, evidently the result of inflammatory action, the larger trunks having been, as he maintains, lined with a coating of purulent matter. Now this is the theory advocated by Mr. Gardner, not from dissections wich he has made, not, in short, from positive facts, but from the following considerations. First, that the constitutional symptoms of phlebitis and those of purpura bear "a close resemblance." Secondly, that in some cases of phlebitis, petechiæ or vibices have been observed. He concludes that it is rather the capillary than the large venous trunks that are affected — that the diseased state appears to extend in some degree to the other tissues—and that purpura is of course an inflammatory disease. Such is a short summary of Mr. Gardner's theory.

It is enough to reply to this hypothesis, that no proof of its correctness is brought forward. Mr. Gardner affirms, that in purpura there is inflammation of the capillary veins, but has not brought forward one case in which dissection has shewn such an affection. In the only cases in which phlebitis existed we are told that there was pus in the larger trunks.

We do not attach much importance to the analogy between the constitutional symptoms of phlebitis and purpura, on which Mr. Gardner dwells. We have witnessed very many cases of phlebitis, and not a few of purpura, but we never perceived the resemblance in question. The symptoms of phlebitis are ordinarily those of typhus; the symptoms of purpura are either inflammatory, or those of debility and deranged health, but seldom actually typhoid. So much with regard to the pathology, which can be firmly and satisfactorily established by dissections only, and not by speculations, be they ever so ingenious.

Mr. Gardner appends two cases which have recently fallen under his observation. They are short and possessed of practical interest; we will therefore take the liberty of extracting them.

"On the 23d May, 1831, I was called upon to visit a young man, aged about 17, a silk weaver. He had a pale and exsanguineous appearance, though not much emaciated, and complained of much debility, accompanied with pain of head, lumbar region, and extremities. His neck, breast, and extremities, but particularly the upper, were studded with petechiæ of various sizes, but none larger than a split pea, some of a red, and others of a purple colour, each with a small white dot in its centre, and remaining unchanged by pressure. None of these spots were to be seen on the mucous membrane of either mouth, nostrils, or eyes. Pulse 104, full and bounding; respiration hurried, and accompanied with a slight cough; skin hot and dry; tongue pale red round edges, but covered in middle with an orange-coloured fur. The gums appeared to be ulcerated, and discharged blood on pressure; bowels open, but the stools were dark and fetid. The petechiæ had made their appearance about six months before I saw him, but up to that time was not prevented from following his usual occupation. He had, however, during that period, several attacks of hæmorrhage from the left nostril, which always ceased spontaneously, without much loss of blood. For a few days previous to my visit, the petecheiæ had been increasing in number, with an accompanying increase of debility. His diet had generally been of a nutritive quality, nor had he been too much excluded from out-of-door exercise.

He was bled to ℥x. immediately after which he became sick, and vomited. A saline purgative was ordered to be taken as soon as the stomach became quiet. Next day I was informed that early that morning an epistaxis took place from left nostril, which was stopped by a neighbouring surgeon, by plugging, after the loss of several ounces of blood. The pain of head was gone; pulse 96, and soft; and expressed himself as feeling better than he had done for some days. The blood taken from the arm coagulated without the separation of serum, and presented a pale red transparent jelly-like appearance, extending to the depth of a fourth of an inch, all under which was coal black. When the clot was broken down a sufficiency of serum was given out. He continued much in the same state under the use of tonics and purgatives till the 31st, when, in consultation with Dr. Hannay, he was found to complain of much headach; skin hot and dry; pulse 112, full and strong, and there was a continual oozing of blood from the left nostril, which was still plugged, and from the gums. There was much thirst and no appetite. From this condition it was judged advisable again to abstract blood, ℥xij. were accordingly taken with very considerable relief. The blood had altogether the same appearance as the former, with the exception of the coagulum being much firmer. Up to the third day from this time, there was great reason to believe that he was recovering. The appetite was considerably improved; the petechiæ were beginning to disappear, and the stools had nearly acquired their natural colour and consistence. These symptoms of improvement, however, speedily gave place to a state of much debility, which continued to increase, accompanied with a low muttering delirium for the two last days, and he died on the morning of 10th June. I regret that I had not an opportunity of examining the body.

About a month afterwards I was requested to visit a sister of his, Mrs. G. aged about 30, of a full and plethoric habit. I found her complaining of languor and lassitude, with slight pain in the lumbar region and thighs. On her breast and arms were scattered a number of petechiæ. They had existed for two days, and were very similar to those of her brother. Her gums were soft but discharged no blood; nor had any hemorrhage taken place from the other passages of the body. Pulse 96, very full. Tongue white. Bowels open. Blood was drawn to the amount of ten or eleven ounces; and she was ordered a dose of sulph. mag. In a few days she had completely recovered her strength, and the whole of the petechiæ were gone. The blood was cupped, and had the buffy coat."

If our readers consider the foregoing cases attentively, we think they will come to the conclusion, that the treatment adapted for one was not adapted to the other. In the first case, the bleeding appears to us to have been decidedly injurious, and, independent of other circumstances, the state of the blood abstracted in the first instance would have made us hesitate before repeating the venesection. This patient was pale and exsanguined, much debilitated, and had been affected for six months. The second case was that of a female of "full and plethoric habit," recently attacked, with a pulse 96 and full. We need not be at the trouble to insist on the difference of circumstances in these cases, nor can we be surprised at the different result.

We conceive that these two cases present a key to the disputes that have arisen on the treatment of purpura. In some instances, we meet with a very plethoric state of system, and in them a moderate degree of depletion is sometimes beneficial. In other instances, and we believe that they constitute the majority, the state of system is any thing but plethoric, and bloodletting is injurious. We saw a remarkable instance of the injurious effects of bleeding. A man had petechiæ on the lower extremities. He did not appear much debilitated, and he was bled without relief. The bleeding was repeated, when immediately the petechiæ increased in size on the left foot, and, although port-wine and bark were immediately given, the foot became black, cold, dead, and subsequently separated at the ankle-joint. We saw another patient with purpura die almost immediately after depletion. We believe that the best treatment is moderate purgation, accompanied with the mineral acids. The infusion of roses, with sulphate of magnesia and the dilute sulphuric acid, with occasionally a dose of calomel, unirritating diet, and the use of tonics, or even stimulants, when the motions become natural and the digestion good, have formed, in our experience, the best mode of treatment. There are circumstances to be attended to, of course, in the individual case—one patient requiring more support, another more antiphlogistic treatment; but we fear that the practitioner who sets out with the determination to treat purpura generally by bleeding will inflict a great amount of mischief.

---

### V. Restoration of Vision, in Cases of Staphyloma and incurable Opacity of the Cornea.

Mr. Nimmo has written an able paper on this subject in our Glasgow contemporary. His object is, to point out the means that have been recommended by German surgeons, and to weigh their comparative merits. We will glance at the operations, which are three in number. The first consists in a removal of a portion of the iris, adherent to the posterior surface of the cornea, in staphyloma;—the second, in the formation of an artificial pupil in the sclerotic;—the third, in the removal of the opaque cornea, and in substituting for it a pellucid cornea, transplanted from one of the lower animals.

Dr. Ammon, of Dresden, was led by considerations, to which we need not particularly allude, to propose the first operation in cases of staphyloma. By means of a hook, introduced into the eye through an opening made in a part of the cornea or sclerotic, at some distance from the most transparent part of the cornea, he proposed to separate more or less of the iris from the cornea, and thus enable the patient to distinguish the light more readily, if not to see. He tried this experiment in one case only, which proved unsuccessful, from chronic inflammation arising, and rendering the part more opaque than before. Mr. Nimmo mentions another case in which the operation has been tried, with indifferent success.

"To this limited experience, I am able to add but one case, which gives little encouragement to a repetition of such attempts. Archibald Gilchrist, nineteen years of age, was admitted a patient at the Eye-Infirmary, on the 12th November, 1832. He stated that he had suffered from small-pox about seven years ago, since which period vision has been totally extinct, a perception of light and shade alone remaining. The right eye was found to be totally destroyed, while the cornea of the left was in a staphylomatous condition. The cornea was white and opaque over three-fourths of its surface, a small portion towards the upper and nasal edge retaining a partial transparency, so that the iris was seen in contact with and apparently adhering to its posterior surface. This case was one which might have been pronounced decidedly hopeless, but it was determined to give the patient a chance of recovering a certain degree of vision, by removing as much as possible of the iris from behind the most transparent part of the cornea. A small incision was made through the lower part of the cornea, through which a hook was introduced, and an attempt was made to lacerate and remove the portion of iris already alluded to. It was found to be firmly adherent to the cornea, and it was not easy to say how much was separated, as some blood was effused and obstructed the view. The operation was not followed by any bad consequence; the wound in the cornea healed rapidly, and only slight pain was complained of for a day or two. In order to promote the absorption of the lacerated portions of iris, the tincture of iodine was given to the extent of thirty drops daily; and after the irritability which followed the operation had subsided completely, the solution of nitrate of silver was dropped on the eye once a day, in order to render the cornea, if possible, somewhat more clear. After a short time the patient left the hospital. The place where the iris had been lacerated was still opaque, and presented a dark mottled aspect. No improvement in *vision* had resulted, but the patient thought that his perception of light was somewhat increased. He was directed to continue the remedies for some time, and it is possible that some further improvement may take place."

This operation, unsuccessful as it has proved in these instances, is inapplicable to conical and racemose staphyloma, so that we need not expect much from it.

The second means—excision of a portion of the sclerotica, was originally suggested by the elder Autenrieth, who performed it on cats with much apparent success. We say apparent; for, as he killed the animals on the fourteenth day, a circumstance which has since been found to prevent the success of the operation, it could not be observed in that brief period. The first operator on the human subject was Dr. J. B. Müller, at that time surgeon to the Ophthalmic Hospital of Reusberg. The patient was a soldier, who, in consequence of the Egyptian ophthalmia, had the left cornea staphylomatous, and the right entirely leucomatous. The operation proved unsuccessful, a white opaque membrane gradually forming in the wound, and the patient becoming as blind as before. The experiment has been repeated by Beer, Himly, and Mr. Guthrie, without success. Dr. Ammon has made several attempts to restore vision in this manner, and Mr. Nimmo has extracted three cases from that author's work. We will give the first and the third, as samples of the difficulties and the results.

"Case 1.—On the 18th September, 1829, Dr. A. made his first attempt at the formation of an artificial pupil in the sclerotic, in presence of Dr. Martini, of Lubeck, Dr. Dieffenbach, of Berlin, and Doctors Hedenus and Hille of Dresden. The patient was a boy, 13 years of age, who had lost his sight soon after birth from ophthalmia neonatorum. The cornea of the right eye was staphylomatous, that of the left was rendered opaque by general leucoma, almost depriving him of even the perception of light. As there was a possibility of doing the patient good, and none of rendering his condition worse, it was determined to form an artificial pupil in the sclerotic of the left eye. The upper eyelid being raised by an assistant, and the eyeball being fixed by introducing a small hook through the conjunctiva close to the edge of the cornea, a semicircular incision was made with a cataract knife through the conjunctiva, near the temporal side of the cornea. The flap was dissected back with the curved scissors, and the bleeding, which was considerable, was suppressed by the frequent application of cold water. The flap of conjunctiva being held back by the assistant with a fine pair of forceps, Dr. Ammon now took a narrow-bladed knife, which he calls a *sclerotome,* and thrusting it through the sclerotic close to the base of the flap, carried it outwards to the distance of four or five lines, and then turned it downwards so as to form a flap. At this moment the patient made violent struggles to get free, the éye escaped from the hook, the lens, with a considerable quantity of vitreous humour, escaped through the wound, and the eyeball collapsed. The flap of sclerotic which had been formed was now removed with the scissors, and was found to bring along with it a part of the choroid coat. The bleeding which followed was not profuse, but continued for a considerable time, and finally yielded to the cold applications which were employed for the purpose of preventing subsequent violent re-action. No inflammation followed this operation. In the course of the next day the eye had recovered from the collapse, and was again distended with the humours. The edges of the opening in the sclerotic were turned somewhat inwards, and the flap of the conjunctiva had shrunk up, so as to leave uncovered a considerable portion of the opening. Round the wound of the conjunctiva was considerable ecchymosis. On the fifth day after the operation, the edges of the wound of the sclerotic began to suppurate, and the opening assumed a longish and narrow form, instead of its former quadrangular shape. A fine silver probe was easily introduced into the wound; this communicated to the patient a disagreeable sensation, and its removal was followed by the escape of some clear fluid, followed by a few drops of blood. Before this took place, the patient remarked that he could distinguish some large body in front of him. He could also perceive the motions of a hand before him, but could not distinguish the form of it. The flap of the conjunctiva had shrunk entirely, and covered no portion of the opening. From this period the wound gradually contracted, a fine membrane, subsequently becoming white and opaque, filled it up, and in the course of a year, the following was the condition of the patient:—'The eyeball was on the whole somewhat smaller than formerly, particularly in its upper portion. The place where the operation of sclerotomy was performed, presented a longish cicatrix of an ordinary aspect, covered by the conjunctiva. There was no unusual vascularity on the site of the cicatrix or around it. The sensibility of the eye to light was neither increased nor diminished.' "

"Case 3.—The patient was a young man, nineteen years of age, whose eyes were affected with conical staphyloma of the cornea, in consequence of an attack of puro-mucous ophthalmia in infancy. It is unnecessary to describe the steps of this operation, which was in almost every respect similar to the other. Immediately after the operation, the patient had a distinct perception of light; the bleeding was easily checked, and the flap of the conjunctiva was readily brought over the opening in the sclerotic. Cold applications were used, and neither inflammation of the eyeball nor of the eyelids followed. Next day, a prolapsus of the vitreous humour was observed, which was clear and transparent. The flap of the conjunctiva had rolled back, and lay at the upper part of the opening. In a few days the surface of the prolapsed portion of the vitreous humour began to lose its transparency; it was covered by a thin, white membrane, to which minute vessels were seen to pass. This gradually became more and more opaque, and became continuous with the conjunctiva, while the humours from behind pressed it forwards, and gave it very much the appearance of a staphyloma of the choroid. The eye remains in this condition, and the patient expresses himself sensible of a considerable increase of perception of light, and has frequently expressed a desire that a similar operation should be performed on the other eye." *

* "Another case, in which the operation was performed by Professor Ulmann of Marburg, is recorded in the second volume of Ammon's Zeitschrift für die Ophthalmologie, p. 123. The result was similar to that of Dr. A.'s cases."

Few patients will be likely to submit to this operation, for the small chance of a modicum of vision which it offers. But it has been attempted to modify the operation, in order to obtain a transparent cicatrix in the sclerotica. Dr. Ammon has proposed to remove a portion of the sclerotica from behind, so that the conjunctiva shall never be opened at the site of the artificial pupil. This, he thinks, may be effected by a needle, with a cutting edge, introduced at some distance from the site of the intended opening in the sclerotic, care being taken to avoid cutting entirely out through the conjunctiva. So far as Mr. Nimmo knows, this operation has never been tried.

Dr. Wutzer, professor of surgery in Bonn, has proposed another modification. He would make the opening in the sclerotic in the original way, then pare away a thin piece from the surface of the cornea at its most transparent portion, and, leaving it attached by a single point, turn it round as in the operation of making an artificial nose, fit it to the opening in the sclerotica, and secure it in its place with a fine suture, in the hope of its adhering and remaining transparent. Mr. Nimmo is not aware of this having been tried, and the operation does not promise much.

The third operation which has been proposed for opaque cornea, is its removal and the transplantation of the transparent cornea of one of the inferior animals. It was suggested by Reisinger, has never been tried on man, and has not succeeded in brutes. D. Dieffenbach, fearing that the transplanted cornea would not unite, offers a modification of the operation. He leaves the original cornea untouched in the first instance, makes an incision *round* it through the conjunctiva, fits into this the edge of the cornea of a pig, and secures it with a fine suture. Should adhesion take place, an incision is to be made through the artificial cornea, and a portion of the opaque cornea to be removed with the knife and scissors.

We need not be at the pains of discussing these suggestions. This joiner's work is not altogether adapted for the eyes of living persons.

### VI. Case of Abscess of the Kidney and of the Cerebellum.

The only method of arriving at accuracy of diagnosis in cases of diseases not attended with prominent and easily intelligible symptoms, is a careful study of particular facts. For this reason we notice two cases reported by Mr. W. Maclean in our Glasgow contemporary for January of the present year. The first is one of collection of pus in the kidney; a disease extremely obscure, and not seldom fatal.

William Semple, aged thirty-eight, farm-servant, of sallow complexion, consulted Mr. Maclean Aug. 22d, 1827, for what was considered a disease of the liver, under which he had laboured for nearly two years. Complains at present of frequent attacks of nausea, with vomiting of bilious matter.—Tongue coated, bowels torpid; dejections of a clayey colour; troublesome thirst, skin hot and dry, pulse varying from 70 to 80 in the minute. Urine voided with pain, and in very small quantity, depositing a muco-purulent sediment. Has a feeling of pain over region of right kidney, and is frequently seized with rigors over the whole body. Has also been troubled of late with a short tickling cough, attended with purulent expectoration, and occasional pains in different parts of chest. Complaints began about two years ago, during which he has consulted a variety of medical practitioners, and has been frequently bled, blistered, and purged. He has also taken mercury to a great extent, but with very little benefit.

He was bled, ordered purgatives, with opiates and digitalis, and the hydriodate of potass ointment was rubbed into the right hypochondriac region. The pectoral symptoms were relieved; the pain in micturition and deposite in the urine continued, as did the pyrexia. Emaciation made progress during the Winter, temporary mitigations of the symptoms took place, but hectic and diarrhœa conspired to carry him off on the 8th April, 1828.

There were tubercles in the lungs—an abscess containing four ounces of pus in the left—the liver enlarged, indurated, with an appearance of numerous small ulcers on its surface—thickening of the mucous coat of the rectum, and contraction of its pyloric orifice—"considerable disease" of the colon and rectum—right kidney adhering to the peritoneum, and containing in its substance about ten ounces of purulent matter—cavity of ureter much enlarged and its coats thickened—urinary bladder contracted.

The symptoms in the foregoing case, which seem referable to the state of the kidney, are the pain in *micturition*, pain in the region of the kidney, deposition of muco-purulent matter from the urine, perhaps the occasional rigors. There are few diseases in the diagnosis of which blunders are more frequently committed, than in that of disease of the kidney. We would wish to direct attention to one or two circumstances. In the first place, pain, referred to the *urethra*, is not an uncommon symptom of disease of the kidney. The patient has all the symptoms of what has been denominated absurdly enough, "irritable bladder"—the presence of calculus is suspected, but on sounding no stone is felt. In other instances there are no very prominent symptoms. The patient has hectic, pain in the back, supposed to depend on diseased spine, or functional derangements of no decided character. Suddenly he is attacked with low typhoid symptoms and coma, and he dies. We have seen cases of either description. The state of the urine should always be carefully attended to. It is mostly albuminous, and commonly acid. Not that albuminous urine is decisive as to the existence of disease of the kidney, but in abscess of the kidney it most frequently contains albumen.

The other case is one of abscess in the cerebellum. It is so brief that we will give it in Mr. Maclean's words.

"Mrs. Watson, a pauper, aged 80, complained for several years of violent continued headach, accompanied with tinnitus aurium, impaired vision, and occasional vertigo, with partial confusion of mental faculties. Although the headach was constant, she had besides occasional darting pains of a more severe nature in the head, and she was subject to frequent rigors, and a constipated state of the bowels. For these symptoms she had the usual routine of practice, leeches, blisters, purgatives, &c., but with partial relief only. For a few days before her decease, the symptoms were aggravated; the vertigo was so great that she was unable to walk without staggering, and nearly falling. She complained of a great weight in her head, so that she could with difficulty raise it from the pillow. In one of the attempts to raise herself she fell forward to the ground in a state of complete insensibility. I found her with stertorous breathing, dilated pupil. slow oppressed pulse, and all the other symptoms of compressed brain. The jugular vein was immediately opened, and twenty ounces of blood drawn off; her head was shaved, and a blister applied to the scalp; but these means were unavailing, she continued insensible, and expired in a short time.

*Autopsy*.—The head was opened fourteen hours after death. On removing the skull-cap, about four ounces of extravasated blood escaped. The dura mater and tunica arachnoidea were preternaturally thickened, and covered with numerous small tubercles about the size of peas; one about the size of a kidney bean was attached to the falx. The contents of these, when cut into, were of a cheesy consistence. Surface of tunica arachnoidea was coated over with a covering of coagulated lymph. Whole of cerebrum unusually vascular; right hemisphere, throughout its whole extent, in a softened state. Lateral ventricles nearly filled with serum. In substance of right lobe of cerebellum, an abscess existed, containing two ounces of purulent matter. Basilar artery was found ruptured, and medulla oblongata coated with lymph."

The only characters which seem to have marked the existence of abscess in the cerebellum, are the long-continued head symptoms, accompanied with *rigors*. It is fair to observe, that abscess in the brain is not always accompanied with rigors. The Editor of this Journal published a case in which abscess of the cerebellum was attended during life with uncontrollable vomiting. On the whole, however, we may lay down a practical rule in diagnosis, which will very often serve us at the bed-side—that if symptoms of disturbance or disease of an organ be attended with fever of a hectic character, and particularly if accompanied by rigors, we may venture to suspect the existence of suppuration in that organ, or that kind of inflammation which is apt to terminate in the formation of pus.

---

### VII. SALUTARIUM IN THE WESTERN HIGHLANDS.

A HIGHLY respectable and well educated physician, long resident at OBAN, in Argyllshire, proposes to accommodate in his own house a few invalids, labouring under dyspeptic, nervous, or other complaints, for which a mild but bracing air, in a most romantic locality, might be recommended during one, two, or three months of the Summer or Autumn—and that on moderate terms. It may be proper to state, that Oban is a small seaport town, on the western coast of Argyllshire, beautifully situated on a bay of the same name, and exactly opposite to the Sound of Mull, the now classic scene of Roderick, Lord of the Isles. Twice a week, three steamers and a stage-coach arrive at and start from Oban—one to and from Glasgow—another to and from Inverness—a third to and from the Islands of Staffa, Iona, &c. while the stage comes in from and returns to Inverary, crossing Loch Awe, and passing through some of the most picturesque scenery in the Highlands. The facilities thus afforded to the invalid of seeing all the most interesting localites in the Highlands, with little expense, while regaining health and strength, are singularly concentrated in the little port of Oban. This part of the Highlands is remarkably healthy, phthisis being nearly unknown, and the variations of temperature being very limited, in consequence of the great predominance of sea over land. There are abundant opportunities for fishing and and shooting in every direction, and the place appears to us (and we have carefully examined its medical topography) to be highly calculated for the restoration of health, as well as for gratifying the senses by scenes of the most romantic and sublime character.

The physician's plan is—"to make frequent excursions with his inmates, of from a day to six or eight days' duration, sometimes by steam-boat, sometimes by land—and frequently in a row-boat or pinnace in the neighbourhood—to all the most interesting scenery in the country—scenes so varied and extensive, that some months might be very pleasantly spent in surveying it."

From some experience, we can confidently assure our professional brethren that a SALUTARIUM of the kind in question, with the advantage of an intelligent physician to attend to the health of an invalid, would be more likely to do good, in a great many disorders, than a tour on the Continent, and that at a comparatively trifling expense. The best season for the Highlands is from the 22d June till the middle or latter end of September—and we strongly recommend the plan proposed by Dr. Aldcorn, of Oban, whom we personally know to be a gentleman of excellent principles, and a physician of skill.

---

### VIII. Dr. Babington.

This venerable physician and excellent man has paid the debt of Nature, at the advanced age of 76, being the oldest physician, in actual practice, in this metropolis. He may be said to have died "with harness on his back," having, as we understand, been engaged in attending others, though labouring under the epidemic himself, till within 24 hours of his end! There were proofs, on dissection, of acute pleuritis in the chest; and this affords one instance, out of hundreds that might be cited, where actual and acute inflammation accompanied the influenza, and was allowed (from theoretical and newspaper oracles) to ravage vital structures till a fatal disorganization took place!

Of the particulars of Dr. Babington's life we know too little for a biographical memoir—indeed few physicians present materials for such compositions, that are of much interest. Dr. B.'s professional career must have been spread over full half a century, and he reaped a rich harvest in that time. Distinguished more by the amiable qualities of the heart than by any striking talent of the mind, Dr. Babington will be more deeply regretted by his personal friends than remembered, or referred to, by his professional posterity. He put on record nothing that will long survive himself. There can be little doubt, indeed, that this most excellent man owed much, if not all his success, to the heart rather than to the head. This excellence of moral disposition, with even an average share of talent, is almost certain to succeed in the end; for when a physician attains a certain age and public standing, his sanction is very generally sought by his professional brethren in all difficult cases, where there is a certainty of honorable conduct on his part. Those MANŒUVERERS who think themselves mighty clever, if they can wriggle themselves into a little practice, at the expense of their neighbours, are short-sighted mortals. Such was not Dr. Babington. He was not the man who, by act, word, or look, would injure the ordinary medical attendant, when called in at any period of the disease. There are but too many whose very nature seems to irresistibly impel them to raise themselves on the shoulders of their brethren, by little, mean, and illiberal tricks.—Every man's observation has furnished him with melancholy proofs of this fact!

That Dr. Babington, in the early and middle periods of life, was a good and even a distinguished practitioner, we can very easily believe; but, during the last fifteen years, our own impression was, that he was very inert and feeble in his treatment of diseases, and that he almost entirely ceased to keep pace with the pathological advances in medical science. This is so generally the case, as life advances, that we must not venture to censure the dead for participating in the failings of human nature. We think, however, that it is the *duty* of the medical practitioner, of whatever denomination, t[illegible] continue to *learn*, while he continues to *practise* his profession. It is hardly fair to derive emolument from a science which we have ceased to cultivate. It must not be said that an old physician has nothing to learn—that his ample experience has furnished him with all the knowledge which can be needful or applicable to his daily pursuits. Quite the contrary. As our faculties fail, or become more obtuse, our diligence, both as regards observation and study, should increase, to make up for the defects of age. It has often been a matter of regret that medical men have clung to the practice of their profession, long after the proper and natural period of retirement; but it must be pleaded in excuse, that old habits are not easily changed or shaken off, and that retirement from the pursuits of a long life is little less than a descent into the tomb before death! As regarded the lamented deceased, however, we think he was just as well qualified to practise his profession in 1833, as at any time during the last fifteen or twenty years. We met him in consultation not many months before his death, and could perceive little deterioration of intellectual power, excepting, perhaps, in the memory. Dr. Babington's professional life must have been a very happy one; for he was never embroiled in any literary disputes, and lived on the most friendly terms with all his contemporary practitioners. His temper was remarkably cheerful, and his manners exceedingly mild. That he never knowingly injured, or even offended a fellow-creature, in this world, we firmly believe—and we have reason to think that, in all the duties of father and friend, Dr. Babington was most exemplary.

His promotion to the Fellowship of the College of Physicians did no honour to himself, and no credit to the College. He must have been past the common range of human existence, when that haughty aristocracy gathered him—not to his forefathers, but to his step-fathers—not to benefit themselves, but to injure the body of licentiates, by snatching from that unhappy class any thing which they considered calculated to embellish it! It was a great pity that Dr. Babington did not imitate the high-mindedness of his senior and contemporary, Sir Gilbert Blane, in disdaining to descend from the head of the list ot Licentiates to be tagged on to the tail of the Fellows! It shewed a great weakness of mind in Dr. Babington, at his time of life—a failing, indeed, which his warmest friends will scarcely be able to negative. But on this disagreeable subject we shall not dwell. Among the titles to our respect and esteem which Dr. Babington has left behind him, few, indeed, who possess any spirit of independence, will enumerate that of "Fellow of the Royal College of Physicians," conferred when the race of life was run, and when one foot was in the grave!

Dr. Babington was one of the very few eminent physicians or surgeons who have deigned to bequeath a legacy to their profession, in the shape of a son. A Hunter, a Baillie, a Cruickshanks, a Halford, a Warren, a Cooper, an Abernethy, cum multis aliis, despised too much the profession by which they rose, to debase any of their offspring by connexion with the ignoble practice of physic! We must, indeed, except our good friend Sir Astley from this censure, for many reasons—one of which, at least, will be considered a *legitimate* one—namely, that he had no son to bring up to his own profession. He has, however, given us so many young Coopers in surgery, besides his talented nephew, that we have no right to complain. Still the circumstance, to which we have alluded, is a very remarkable one, and serves, we think, to prove, incontestibly, that medical science is not so much respected by its most illustrious professors as it ought to be!

---

### IX. ON HYSTERALGIA, OR IRRITABLE UTERUS. BY DR. D. DAVIS.

IN that very excellent work now publishing in parts by Dr. Davis, we find an article on hysteralgia, which we deem worthy of notice. The term was first given to the complaint by the late Dr. Gooch, having previously gone under the names of "painful menstruation"—"uterine irritation"—"chronic inflammation of the uterus," &c. Dr. Gooch's description of hysteralgia is adopted by our author, who next proceeds to criticise the theory of Dr. Gooch, who considered hysteralgia as a purely functional disorder, unconnected with inflammation, and not ending in any change of structure.

"This plausible theory, (says Dr. D.) of an exquisitely painful disease, without the co-existence of inflammatory action of the affected organ, must at best, in the present state of our knowledge, be considered doubtful as to its correctness. It is not even certain that we are yet acquainted with all the possible forms of inflammation, so as to be competent to assert broadly and emphatically that this or that variety of inflammation should have a natural and necessary tendency to end in disorganization of structure. It is not easy to conceive of certain forms of rheumatalgic affections, for example, such as lumbago and sciatica, without connecting with them the idea of an inflammatory condition of the tissues principally concerned; and yet on that account, who ever supposes that such inflammatory actions have a natural and necessary tendency to end in malignant disorganization of structure? If muscular fibres be a constituent tissue of the uterus, why might not such fibres become the subjects of a painful inflammatory affection, a truly rheumatalgic affection, without being followed, any more than in the other case, by a malignant disorganization of structure? It is well known that the uterus is not unfrequently the subject of very painful states, occasioned exclusively by functional causes, as we see constantly exemplified in cases of disordered menstruation, leucorrhœa, etc.; but does it necessarily follow, that such morbid conditions are essentially independent of all inflammatory action? Or rather, is it not demonstrable that of some of them, at all events, inflammatory action is an essential attribute? And yet we find that such painful states, such demonstrably inflammatory affections, may be sustained for many years without producing malignant disorganization of structure. The limits subsisting between the phenomena respectively of irritation and inflammation, are not yet established with sufficient precision to enable us to determine with perfect confidence under which of these heads some doubtful forms of disease should be classed. Many diseases, loosely attributed to irritation alone, are often characterized by symptoms which a more accurate diagnosis would enable us at once to ascribe to actual inflammation. In the description of the irritable uterus as above quoted, we encounter several symptoms which are known to be constant accompaniments of inflammatory action. All the occasional causes of the disease, as enumerated by Dr. Gooch, as well as the greater number of its essential symptoms, would seem to lead to the supposition of a proximate state of parts, if not actually inflammatory, at least one of no inconsiderable vascular congestion; for in addition to a morbid state of the nerves of the affected organ, which is not disputed, there is also unquestionably a morbid over-distension of its blood-vessels during the presence of this disease: and this is, after all, the point of greatest importance practically to attend to, inasmuch as it bears immediately on the principal feature of the treatment to be adopted. The designation given by Dr. Gooch of 'the irritable uterus' to the distressing malady which he has here so admirably described, is therefore so far objectionable, as it leaves out of view one of its original, and perhaps its very principal constituent, viz. A PAINFUL OVER-PLENITUDE OF A PART AT LEAST OF THE INTERNAL ILIAC AND PUBIC SYSTEMS OF BLOOD VESSELS. Such a condition of the blood vessels in question is more or less an obvious result of the occasional causes by which the disease is represented as being most frequently produced. It is promoted and exasperated by whatever exertions or other causes which may be supposed calculated to increase the over-distension of the uterine vessels. The author recollects the case of a painful affection of the right foot, which was incurred by a gentleman some eighteen years ago by over-exertion in walking. At first the pain was considerable, and greatly interfered with the gentleman's pursuits; which required much personal activity. It was subject, like that of the irritable uterus, to occasional abatement, according to the degree of rest which could be afforded to the affected limb, and to more or less exasperation, according to its exposure, which, indeed, was unavoidable, to more or less of walking exercise. It was, however, at no time so severe as to render walking totally impracticable. For this reason the case was almost entirely neglected in the beginning. It consequently became a chronic affection, which, although it gradually abated of its original violence, has never altogether ceased to occasion inconvenience. Now the reader will easily recognise something of analogy between the occasional causes respectively of the irritable uterus and of the lamed foot. If the painful effects be not of a nature to be identified with a state of inflammation in the one case, it would of course be quite proper to dispute its existence in the other. On the other hand, an over-extension of tissue in the one case might be expected to produce a similar result as to proximate effect to what is known to take place in the other. In the foot case a state of exhausted power was followed successively by an over-extension of fibres and a slow sub-acute inflammation of the injured tissues. Of the fact of the latter result, the author has most abundant reason to be quite certain. But why admit such results in the one case, and deny or totally overlook them in the other? Of the foot case the proper treatment undoubtedly would have been the application of a suitable number of leeches to the part, and the immersion of it for an hour or two afterwards in hot water, or the assiduous application for an equal length of time of hot fomentations to the surface, followed up by a repetition of the same practice on the next, or on an early day, subsequently; giving also to the limb the benefit of two or three weeks' most perfect rest. But it may be very well asked, whether the idea of exclusive irritation could be supposed so directly to lead to the proper practice in such a case, as that of inflammation, or of that even of congestion of the vessels of the part consequent upon the application of the previous injury. The author thinks not. For the same reason, he therefore thinks that the new designation of Dr. Gooch, as applied to the morbid condition of the uterus, which in many respects he has most faithfully described, may have the effect of leading practitioners to an inert and procrastinating practice. About two years ago a case occurred within the cognizance of the author, very well suited to illustrate the tendency of a name to impose upon a weak mind, in an affair precisely of the kind, or rather in the instance of the very disease which we are now describing. Mrs. S. of B. Crescent, a very delicate lady, of about thirty years of age, and the mother of a numerous young family, had been the subject of much uterine irritation for about eight months, for the relief of which nothing very efficient had been done by her ordinary medical attendant. The husband, without giving any intimation to that gentleman of his intention, requested the present reporter of the case to pay his lady a professional visit, and to favour him with his opinion of the nature of her malady, and of its probable issue. The neck of the uterus was found exceedingly painful and considerably swollen; but without structural disorganization. The patient was greatly attenuated, and very pale and spiritless. The case was reported as one of no urgent danger, but nevertheless one involving some ultimate risk, if the present symptoms, which were represented as those of a peculiar variety of inflammation, could not be subdued. On being requested to see the patient again, he suggested the propriety of his being met by the family medical attendant. But that person was unappeasably offended at the husband for requesting another opinion without previously consulting him and without his consent, and declined all further attendance on the case. In a short time, however, afterwards, upon learning the author's opinion, he took great pains to represent it as being totally unfounded; adding, that if it should be acted upon, the practice would soon prove fatal to the unhappy patient. The neck of the uterus, it has been already stated, was considerably swollen. With the aid of a speculum, it was seen to be also in a state of intense superficial inflammation. All its vaginal portion was of a vividly red colour, similar to that of external genital surfaces when become the seat of a recent gonorrhœal affection. A quantity of viscid mucus was seen distilling from the uterine orifice. Little intimidated by the angry oracular prognostics of his predecessor, the author hesitated not to order four leeches to be forthwith applied to the orifice of the uterus. This duty was performed by the very intelligent midwife of the Maternity Charity, whose useful services in this respect he has already had occasion to notice. He was induced to limit the number of leeches to four, in consequence of observing how intensely the vaginal part of the organ was charged with blood. The quantity of blood obtained amounted to at least ten ounces, and the abstraction of it was almost immediately followed by the happiest results. After an attendance of about three months, during which the application of between four and six leeches were repeated four or five times, the author on retiring had the pleasure of leaving his fair patient in a state of much comparative comfort, of almost total freedom from the distressing pain of the uterus which had recently embittered her existence, and in other respects rapidly recovering her former health and strength. There was in this case very probably the irritability of the uterus, which had been represented by the family attendant as the patient's peculiar malady, but which had been in no degree mitigated by the soothing and strengthening medicines exhibited by him for its relief; but there was also most unquestionably much positive inflammation of the vaginal portion of that organ, the removal of which, by the depleting measures already described, made way for the eventual subduction also of the accompanying irritability. In the case of 'THE IRRITABLE UTERUS,' there is a period of recency and comparative acuteness of symptoms as certainly as there is in those of the irritable tumour of the breast, and of painful affections of knee and ankle-joints from over-extension of their ligaments; and there is little doubt but early and efficient vascular depletion would be quite as beneficial in all cases of the former, as they would probably prove in either of those of the latter. But would the hypothesis of a mere irritableness of the part in any one of these cases directly lead to such a practice? Again, the author thinks not. He accordingly finds local bleeding placed by Dr. Gooch under his second head of remedial measures; whereas the supposition of an over-fulness of the vascular system of the affected organ would naturally point to the relief of such a state AS A FIRST MEASURE. But if the disease be one of irritation and not of inflammation, nor of any condition of the parts allied to that of inflammation, why bleed at all? Because probably the utility of the practice had been fully ascertained by experience before the theory of the irritable uterus had presented itself to the mind of its talented propounder."

From the above observations it is evident that the author considers vascular depletion as forming the main feature of the treatment. But general blood-letting, he thinks, can rarely be necessary. The disease is local, and local abstraction of blood from the os uteri, he avers to be the best remedy. The complaint is usually concealed for a long time, and consequently the cure is rendered thereby tedious. The application of four leeches to the os uteri will generally secure the abstraction of eight or ten ounces of blood, and be succeeded by relief of the symptoms. The second most important measure is the horizontal position—and these two means, if early had recourse to, would soon reduce the disease; but when become chronic, then the recovery is tedious.

"There is however, one point of practice, in reference to this form of the disease, to which the reader will do well to pay particular attention. The subjects of "the irritable uterus" are not always unsusceptible of impregnation. On the event of conception taking place during a period of remission of its most urgent symptoms, the medical attendant should then more than ever, and especially during the earlier months of gestation, insist upon the strictest conformity to his precepts in respect to the observance exclusively of the horizontal position. The action of gestation introduces a great change into the uterine system. During the last four months it places the uterus in a situation to be in a great measure secure from the attacks, if not altogether beyond the reach of some of the most influential occasional causes, of the disease. On the completion of the process of parturition, the patient may indeed be said, in reference to her former complaint, to have the opportunity of commencing a new life. If, during that period, she could be induced to keep her bed, in the most literal sense of that expression, for six weeks or two months, she would almost certainly secure herself against a relapse of her complaint subsequently to her confinement. In consequence of the prodigious development of parts interested in the business of gestation, nature is observed to exhibit a power of self restoration and adjustment during the puerperal state, which at no other time nor under any other circumstances does she seem competent to exert. Hence, in cases of moderate prolapsion of the uterus incurred by forward conduct, during one confinement, a perfect cure may frequently be obtained by the patient confining herself to her bed, and maintaining rigidly the horizontal position for at least five or six weeks subsequently to her next delivery."

Anodynes are necessary, and opium is the most efficient; but as it too often confines the bowels, and checks the biliary secretion, hemlock, hyosciamus, &c. combined with camphor, become necessary as substitutes. Battley's laudanum thrown into the rectum will often succeed, where it is found to disagree if taken into the stomach. As an aperient, Dr. Davis recommends sulph. magnes. in infus. rosar.—castor oil—electuary of senna—sulphur. In some cases of irritable uterus, accompanied by obesity, Dr. D. has seen good effects from mercury, as an alterative.

---

X. Observations on the present State of Pharmacy in Ireland, &c. By Denis Phelan, M.R.C.S. of London. Clonmel, 8vo. pp. 160.

Mr. Phelan is an able and assiduous labourer in the field of medical reform, and well deserves the support of those who wish to see our professional institutions purged of what requires removal. The master evil, the prime mischief, the very heart's disease of almost every one of them, is the connexion which subsists between the pursuits of a noble occupation, like that of medicine, and the debasements of mere mercenary traffic. Science and trade can never be married; the very essence of their natures is antipodal; and as well may we expect to see a union between the eagle of the sun, and the crawling tortoise of the fields. It is an ignorant, if not a downright criminal enactment of legislation, which has ordained such an unholy contract, and no honourable man, certainly none who has any regard for the dignity of the profession, would lend support to so unworthy a connexion. Let us hear some of the clauses of the Act of the Irish Apothecaries' Company, and inquire for a moment or two into their proceedings, before we condemn. The first clause states that "There shall be within the City of Dublin, one Company of judicious Apothecaries, well skilled in preparing and compounding of medicines; and there shall be a hall amply supplied with medicines of the purest quality, prepared under the inspection of persons well skilled in the art and mystery of such preparations." The sixteenth clause empowers them "to make and constitute bye-laws and ordinances, for and relating to the affairs and government of the said Company," and the twenty-second authorizes them to examine all applicants for an Apothecary's licence, "as to his qualification and knowledge."

Mr. Phelan argues, and with much plausibility, that neither these, nor any other of the clauses of the Act, invests the Apothecaries' Company with the power or privilege of instituting a regular curriculum of education, obligatory on all who apply for a certificate; or of instituting courses of lectures of their own, and appointing Professors. Indeed, the very circumstance of the Hall having only within the last five years presumed to exercise such a privilege, is almost proof enough that the right was not clear or indisputable. From the year 1791, when the Act passed, till 1827, no attempt was ever made to step beyond the limits of simply selling drugs, and of examining apprentices and master apothecaries in their knowledge of pharmacy and materia medica; and it appears from their own admission, that their mercantile concern was in a most flourishing condition; for the shares originally £100. each, had risen to six times their value, and some of the worthy members had cleverly contrived to have five or six of these in their own possession, and it is even gravely offered to be proved before Parliament that "a person who never was an apothecary had eight or nine of these money-making shares, whilst many of the most respectable and talented apothecaries in Dublin were in vain endeavouring to get one." In 1827, however, the Hall seems to have wished to announce publicly the marriage of the trade of the druggist with the science of medicine. The preceding thirty-six years had been as it were, the period of courtship, during which the bridegroom had very successfully contrived to make an exceedingly comfortable provision for the establishment of his house; he was now in sooth a thriving man, had vastly increased his traffic, and deemed himself as fit as any one to be a patron of science; especially as it might probably redound to the credit of his purse, as well as of his taste. He therefore ordained an academical course to be followed by the aspirants for the honors of apothecaryship; the extent of this course was attendance on two classes; one on materia medica and pharmaceutic chemistry, and the other on medical botany; even here was the favouritism of trade exhibited.

At no University is there a course of lectures on what is styled pharmaceutic chemistry; the student was therefore obliged to attend two separate courses, one on chemistry, the other on materia medica, unless he selected the short single one on chemical pharmacy delivered by the Professor whom the Hall had recently appointed, but whose ticket was not recognised at any place, for a medical or surgical degree. In 1831 the Directors published other regulations, extending the course of lectures on chemistry, materia medica and pharmacy, and adding thereunto one on anatomy and physiology, and one on the practice of medicine. All this bears the front of an enlightened and well-informed legislation; and so it really was, if not tarnished by some disgraceful jobbing which was carried on at the same time. By the 22d clause it is provided "that no person shall open a shop, or act as an apothecary in Ireland, unless he has served an apprenticeship, and has been examined as to his qualifications and knowledge by the Hall Directors, and that all offenders are to be prosecuted." The interpretation of this must be obvious to a child; and surely it is equally manifest, that if it can be proved that the Company has wilfully compromised and neglected their duty, and permitted ignorant and uneducated men to practise the business of an apothecary, men who had never served an apprenticeship, and who had never been subjected to any examination; and that the Company afterwards admitted these very men to all the privileges and rights of regular apothecaries, upon paying the sum of £20.; they virtually encouraged the very abuses which they were incorporated to prevent; and while they put money into their own pockets, seriously injured the regular apothecaries who had complied with all the provisions of the Act. Naturally enough there was a loud and general clamour of indignant hostility against such barefaced injustice, and meetings were held in different parts of Ireland to petition Parliament for some changes in the Apothecaries' Act. The Hall Directors' appear to have been aware of the feeble stability of their claims, for they invited a general meeting of the apothecaries in Dublin; but their conduct both before and at this meeting did not indicate any sign of liberality or justice.

Unfortunately, the Dublin apothecaries, (who by-the-bye are alone eligible to the Directorship) did not act in unison with the apothecaries from the other towns and from the country, else, in all probability, the voice of calm unanimity might have gained for them the object of their desires, namely, that of wresting the monopoly from the Directors, and of converting the Hall, from an irresponsible self-elected body of men, to an enlightened and responsible Association.

A committee indeed was formed, and a deputation sent to London to have an interview with the Government on the subject of the usurpation of the Company. The petition was entrusted to the care of Mr. Donovan, under the firm belief that this gentleman was quite favourable to the views and wishes of his constituents; but far otherwise proved to be the case, and Mr. Phelan openly charges him with disingenuous dealing, and truckling to the Directors. The case however is stated to be under the consideration of Government at the present time, and we may perhaps look forward to some speedy change in one, if not in more, of the medical establishments of this country.

Let it not be supposed by any of our readers, from the remarks we have made relative to the powers assumed by the Irish Apothecaries' Company, to extend the courses of required professional education, that we are in any degree friendly to a low standard of qualifications. Far from it: it is not to the propriety of the thing itself, so much as to the right of a particular set of men, incorporated originally, it would seem, rather as merchants than as guardians of an exalted profession, that we object; and even in objecting, we wish it to be remembered, that we are but echoing the sentiments of our author, until we have an opportunity of hearing the arguments of the other side, and of reading their apology. This may perchance prove exculpatory, and may even rebut the averment of the Irish College of Surgeons, that "the Hall had no right or title to institute professorships; that it would be fitter for the apothecaries to be minding their shops, than aspiring to the higher branches of professional knowledge; that it was the unanimous resolve of the College in all ways and on all occasions to oppose such an assumption." Of such an import was the reply, says Mr. Phelan, to a petition of the apothecaries addressed to the College, that the lectures of their professors might be recognised at Stephen's Green.

It is truly distressing to hear of such bickering and quarrels among medical men, but it requires not a prophet to tell us, that until medicine is divorced from drug-selling, and until a high and uniform standard of medical qualifications be admitted, there must continue to subsist the abominable practice of envy, jealousy, and back-biting, which has tended so much to degrade and vilify our profession. The subject might occupy a volume of lamentations, but our limits preclude us from indulging for the present.

---

### XI. DISCHARGE OF WORMS FROM VARIOUS PARTS OF THE BODY.*

John Alexander, aged 10 years, was for nearly a year in a delicate state of health; although his appetite continued pretty good, he had much wasting of flesh and general debility. About eight months since a tumour arose over the epigastrium, which after being poulticed for some days, burst and discharged, with about two ounces of pus, a white worm half an inch long. In a few days the abscess healed. Eight or ten days afterwards a second tumour arose about

* Extract of a letter from Mr. C. Neilson, of Killala.

three inches distant from the first, on the right side of the chest, which after some days also burst and gave exit to another worm. It is needless to particularize the different instances, suffice it to say, from the time of the first worm being discharged until I first saw him, which was an interval of two months, five worms had made their appearance. They were all similar to the first, and lived for a few hours after their discharge. When I saw him the integuments of the right cheek and eye were excessively swollen, and in the course of a few days another worm was discharged from the upper eyelid. I recommended different medicines for the space of six weeks, but the formation of abscesses on different parts of the trunk and extremities proceeded, and altogether about twenty worms were discharged, principally from the right side. At length two grains of calomel were given every night until the gums became affected, and convalescence shortly afterwards took place, I shall not pretend to say whether from mercurial influence, or from the produce of the original nidus having been exhausted. The boy has been now for nearly three months quite well, his health and strength being completely re-established. The worms appeared to be ascarides. None were, however, at any time observed to be discharged from the intestines, nor were the bowels irregularly affected.

I cannot account for their formation: whether the first deposition of eggs had been made by some means under the external skin, or whether a worm had perforated the intestine, and at length made its way to the surface. I incline to the latter opinion from the boy's previous ill-health. I could in a few instances trace a reddish line from one abscess to the other, this was, however, after the new abscess appeared; the patient himself felt no uneasiness in the part, nor had he any idea where the next abscess would form until it appeared externally.

---

XII. Observations on the Testicles. By James Russell, F.R.C.S. &c. &c. Edinburgh.

Montesquieu, in his Lettres Persanes, makes the following remarks on compilations and compilers.

"De tous les auteurs, il n'y en a point que je méprise plus que les compilateurs, qui vont de tous cotés chercher des lambeaux des ouvrages des autres, qu'ils plaquent dans les leurs, comme des pieces de gazon dans un parterre: ils ne sont point au dessus de ces ouvriers d'imprimerie qui rangent des caracteres, qui, combinés ensemble, font un livre où ils n'ont fourni que la main."

Voltaire seems to have participated in Montesquieu's horror of compilers, for in his Satires he enumerates as one of the contemptible exploits of the Abbé Trublet, that—

"Il compilait, compilait, compilait."

We suppose that the wrath of these able men was not so much directed against professed compilers, as against those soi-disant original writers, who attempt to palm off old wares and stolen goods, as their own indisputable and legal property. Compilations are undoubtedly useful in their way and in their place, but when they are launched and sail under false colours, they are little better than pirates, and deserve to be fired into as such.

We have every respect for the author of the work before us, and believe him perfectly incapable of pretension to more than is justly his own. In a very modest preface he informs the public that he has preferred giving cases from acknowledged authority, rather than from his own, and the observations are an extension of lectures which he originally delivered when Professor of Clinical Surgery in the University of Edinburgh. But we question Mr. Russell's judgment in giving these observations to the world. If presented with the expectation of profit, we fear he will be disappointed — if of fame, we are afraid that like the spirits of the vasty deep, she will not come when she is called.

We have looked through this little volume, and have found it little more than a compilation. It would require an original and a bold mind, and a vast experience to venture on this subject so soon after the publication of Sir Astley Cooper's work. The sort of style and of matter that suits a dictionary is not adapted to a monograph, and here it is that we think our author has sinned. Had he written the article in a terse manner and more condensed form for a cyclopædia, it would have done sufficiently well, but we fancy that, as it is, the result will be less satisfactory.

As we hate finding fault, we shall leave the ingracious task of criticism, and endeavour to select a few passages for the amusement or instruction of our readers. Speaking of anomalies of the testis, Mr. Russell mentions the following facts.

"Several other anomalous cases are recorded. In one, the scrotum was corrugated, and the testicle elevated upon the blowing of the East wind, which, in general, is a cause of languor and relaxation. In another the testicle ascended into the inguinal canal, whenever the party was in company with women.

But the most curious deviation from the ordinary course of descent, is when the testicle, instead of descending through the inguinal canal, which is its natural course, accompanies the femoral vessels in their progress under Poupart's ligament, making its appearance at the bend of the thigh. Mr. Arnaud gives several instances of this singular variety. The most instructive case is detailed at considerable length. An officer, about forty years of age, consulted Mr. Arnaud respecting a swelling in the bend of the thigh, which was taken for a hernia. Upon an accurate examination of the case, however, Mr. Arnaud satisfied himself that the swelling was not a hernia, but a misplaced testicle. He adduces three reasons in support of his opinion. 1*st*. That the officer had not a testicle in the same side of the scrotum. 2*d*. That the swelling had the form and consistence of a testicle, the appearance of the spermatic chord alone being sufficient to distinguish the case from a case of crural hernia. 3*d*. That pressure produced exactly the same sensation on this as on the other testicle. Mr. Arnaud gave a most judicious advice, that the patient should, now that the testicle had passed out of the abdomen, treat it as he did the other testicle, by doing nothing."

We fancy that the East wind is a cause of languor and relaxation, or just the reverse, according to circumstances. If we look at peoples' noses during an Easterly blast in London, we may venture to conjecture from their blue and pinched condition, what the state of the scrotum may be. Mr. Russell makes a remark in which either his English or his observation must be at fault. "The right testicle," says he, "is said to be more frequently affected with scirrhus, and the left with varicocele. But the truth of this remark requires confirmation." The truth of the first assertion does so, but the second is the expression of a fact so notorious that we feel surprise at any surgeon doubting it.

It is a proverb that there is nothing from which nothing is got. We were not aware, for instance, that young debauchees were called God's geldings, and we do protest, at all hazards, against the propriety of the term; if geldings at all, they are either their own or the devil's. The instinct by which they are detected is curious, indeed, as our American friends say, important, if true. But we will let Mr. Russell tell his own tale.

"Though it is a well-known fact, that young men of fashion, who indulge their amorous propensities to excess at an early age, lose the power of procreating sooner than the more continent, and are familiarly distinguished by the quaint appellation of God's Geldings, I never have been able to discover any exterior marks of discrimination, to distinguish these from other men; but the ladies, who are more discerning in such matters, predicted, in a district of country with which I am acquainted, of six gentlemen in the prime of life, that they never would beget children. The event verified the prediction, as all the six were married, and every marriage proved barren."

*Seminal Emissions.*

Mr. Russell makes some remarks on involuntary seminal emissions, a very distressing, serious, and often incurable complaint. There is no surgeon in extensive practice in London who does not see very many of these cases. Mr. Russell observes that the complaint may be produced in two ways: by two great continence, or by a high degree of morbid irritability and weakness. The latter is the most frequent and the most important, for the treatment of the former is obvious and usually effectual. In point of fact, the same remedy, sexual intercourse, is applicable to both cases, though the degree to which it is employed must, of course, vary.

"Seminal emissions, in persons enjoying health, are preceded by a full erection of the penis, although no emission takes place till the subsequent stimulus of coition excites the requisite actions in the other organs of generation. But in persons afflicted with morbid irritability, the case is widely different, since in them emissions take place without a full erection of the penis, and without the additional stimulus of coition. The general diminution of power in the generative system, the inseparable concomitant of morbid irritability, occasions both a failure on the full erection of the penis, and a want of retention in the secreting organs, thereby deranging the performance of their functions."

We need not take up our reader's time by dilating on the principle of treating such a case by enjoining sexual intercourse. We have seen one or two instances of complete success, but we have also found that it is extremely difficult to prevail upon some patients to adopt the prescription. We saw a gentleman last summer, who, after struggling for two months to muster sufficient courage, withdrew himself from our care, in consequence, we have no doubt, of a feeling of shame at his want of determination. We have at this moment under our charge another gentleman, who cannot be persuaded to try the experiment.

We have found that this morbid disposition to seminal emissions, conjoined, as it generally is, with more or less deficiency of the vis virilis, is too often owing to the habit of masturbation. The testes not unfrequently waste in these cases, and the patient becomes nearly, if not altogether impotent. But this is not all. We have seen these symptoms attended or succeeded by the most excessive and intractable irritability of the bladder. We say irritability of the bladder, but we merely mean by that expression a certain assemblage of symptoms, without being satisfied of the exact structural or functional alterations on which they depend. The patient has most of the symptoms of stone in the bladder, even, in some instances, bloody urine. Conjoined with these symptoms, there is usually pain in the region of the kidney, and, in some cases, albuminous urine, rendering it more than probable that the kidney is affected. Never having witnessed a dissection of a patient affected in this manner, we cannot pretend to be acquainted with the exact nature of the case. We are sorry to say that we have found no treatment permanently beneficial.

Our author's methodus medendi of the seminal emissions depending on or connected with morbid irritability, appears to be judicious.

"The second variety, as it depends upon weakness and irritability, is much less tractable, and often baffles the most judicious treatment for a great length of time. The indication of cure, so far as diet is concerned, is the direct contrary of the other variety, since abundance of nutritious food, to communicate strength and invigorate the system, is an indispensable requisite of the medicinal treatment. Stimulants of all kinds must be carefully avoided, or used in great moderation under special circumstances when great languor and lassitude prevail. The patient should also be abstemious in liquids. He should not indulge in habits of a relaxing nature. Instead, therefore, of sleeping on a soft feather-bed, he should lie on a firm mattrass, with the air of the room at a moderate temperature, and he should not allot many hours to sleep. He should pass much of his time in the open air in a cool atmosphere, take frequent and moderate exercise, neither used so long, nor pushed so far, as to occasion fatigue. Cold bathing, too, is a useful auxiliary. Even local cold bathing, frequently repeated, is of advantage; for which purpose, a vessel of cold water may be placed at the bedside, and a wet cloth applied occasionally to the testicles. Great attention should be paid to preserve the general health unimpaired; and, with regard to particular medicines, there are none more serviceable than the different preparations of iron.

Besides the above practices, which relate to the state of the body, it is of great consequence to attend also to the state of the mind. With this view, the patient should banish from his thoughts every lascivious idea, and abstain from reading any book addressed to the imagination on matters of love. And as patients affected with these complaints are apt to despond, and to be troubled with low spirits, every recreation should be encouraged, to prevent them from thinking of their own situation, and, if possible, to keep them amused, or at least to keep their minds occupied with objects which engross the whole of their attention; the patient, at the same time, courting the society of lively and agreeable company. By uninterrupted perseverance in this mode of treatment for a sufficient length of time, the patient may reasonably entertain hopes of obtaining a radical cure."

We think that the great art and great difficulty of treating these cases consists in giving tonics to a certain point and no farther. If we give too much, if we stimulate, the patient becomes feverish, and is rendered worse; if we deplete, he is rendered worse also. Early hours, attention to the state of the bowels and of the digestive organs, relaxation of mind, country air, the shower-bath cautiously begun and judiciously persevered in, with properly regulated sexual connexion, are the means which we have found most beneficial.

*Enlargement of the Scrotum.*

The English public have been lately rendered familiar with this curious affection by the celebrated and unfortunate case of Loo Choo. The complaint is precisely similar in kind to the Barbadoes leg, and essentially consists of deposition of lymph, but little vascularized, into the cellular membrane. It is occasioned, as in the instance of the Barbadoes leg, by repeated attacks of slight inflammation, each of which leaves a fresh deposition.

Mr. Russell observes that it is endemic and very prevalent among the Bambara nation, on the coast of Guinea, and there it is regarded as a mark of nobility. When the patient goes out to ride, the scrotum is supported on a bowl placed on the pummel of the saddle; and when of the largest size, has a sheet passed over the shoulders, and dragged along the ground, when he attempts to walk. Our readers will recollect, no doubt, Goldsmith's humourous story of the traveller entering a church in Switzerland, where all the congregation had double chins.

Mr. Russell observes that he is aware of only two cases in which the disease originated in Europe. One occurred in the practice of Mr. Liston, of Edinburgh—the other in that of M. Delpech, of Montpellier. Both were related in this Journal. In the West Indies, though long confined to the Island of Barbadoes, it is now extensively diffused among the other islands.

*Gonorrhœa and Inflammation of the Testis.*

Mr. Russell has made some remarks on this head, that we notice for the purpose of criticism.

"The swelling of the testicles upon the cessation of the discharge, suggested the idea of the affections being vicarious. The transference of disease alternately to the testicles and urethra was also referred to metastasis, or the translation of morbid matter from the one to the other. This doctrine of metastasis, however, is not applicable to those cases in which the discharge from the urethra ceases before the swelling of the testicle appears, since, as there was not any collection of matter to be absorbed from the urethra, none could be transferred to the testicles. The same argument controverts the doctrine of the inflammation being propagated from the urethra to the testicles along the course of the vasa deferentia. For, if inflammation were extinct in the urethra, it never could be the source of a new attack in the testicles. It is further evident, that when the inflammation of the testicles precedes the affection of the urethra, it must depend upon a very different principle. All we know upon the subject is, that a morbid poison is applied to the penis, and that, after lying dormant for some time, its effects become apparent in the production of various distressing symptoms. And that all the affections of the urethra and testicles are the consequences, more or less direct, of its influence upon the organs of generation. The existence of an intimate relation between the urethra and testicles is proved by a number of cases of daily occurrence, in which the relief of the urethra alternates with the suffering of the testicles, and *vice versâ*. But this alternation, though general, is not universal, and therefore cannot be received as a fundamental law of the system. There are two sets of cases equally incompatible with the doctrine of reciprocity. First, when the inflammation of the testicles does not commence till the discharge has continued for some time, and the discharge, instead of abating, proceeds without interruption in its ordinary course, the affection of the testicles following the same course, whereby a direct sympathy is established. Secondly, when, in the case of a testicle swelling upon the suppression of a discharge, the swelling does not subside upon the restoration of the discharge. Both these cases are alike inconsistent with the truth of the doctrine.

There is, however, sufficient evidence that the urethra and testicles are under the influence of the most delicate sympathy. A chronic obstinate swelling of the testicles often depends upon the presence of a stricture of the urethra, which passed unnoticed, as it neither had excited pain nor produced any difficulty in passing water. The testicle subsides upon the cure of the stricture. But this mode of cure cannot always be employed with effect, as the introduction of a bougie often excites intolerable irritation in the urethra, or affects the testicles, and prevents the continuance of the practice."

Mr. Russell has shewn, satisfactorily enough, that hernia humoralis, is not dependent, as is frequently imagined, on the metastasis of inflammation from the urethra, because it most frequently occurs whilst free discharge is taking place from that canal. But we do not think him so successful in disproving its dependence on extension of inflammation along the mucous membrane lining the vasa deferentia. The reasons which he urges against this supposition are, first, that hernia humoralis occurs when the urethral discharge has ceased; and, secondly, that it may precede the appearance of this discharge. To this we reply, that in the very great majority of cases, inflammation of the testis occurs during the existence of urethral discharge, or immediately after its cessation or diminution—that it very seldom succeeds it at any considerable interval, and still more rarely precedes it. To this we may add the fact, that, in most cases of gonorrhœal hernia humoralis, the parts chiefly affected are the vas deferens and its continuation, the epididymis, parts which, of course, would be chiefly affected by continuity of inflammation. Mr. Russell's theory, that hernia humoralis is a consequence of the application of a morbid poison to the penis, will not bear examination, or at least amounts to nothing more than a vague assertion. A morbid poison can, so far as we know, affect a remote part in three ways only—by continuity of tissue—by metastasis of action or inflammation—or by absorption, and the production of secondary symptoms through the medium of the constitution. The two first are denied by Mr. Russell, and we must presume that he supposes the third process to occur. We need only say that there is no proof of its occurrence, and many weighty reasons for disbelieving it. This is not an idle question, but bears upon the treatment of gonorrhœa. If stimulating means are adopted to arrest the inflammation of the urethral mucous membrane during its height, and at a time when it is attended with fever, or much local pain; if, in short, large doses of cubebs or capivi be given, or wine drunk, or violent exercise taken under such circumstances, we incur a considerable risk of causing the inflammation to extend along the vas deferens to the testis. On the other hand, it is obvious that, if it be not mere metastasis, it is idle to think of relieving hernia humoralis by means applied to the urethra to increase the discharge.

We have one remark to make upon the idea, that inflammation of the testis not unfrequently depends on stricture, and may be cured by curing the latter. This is pure fancy. It is true that, with chronic inflammation in the urethra, we are likely to have extension of the inflammation along the vas deferens, and thickening of it and the epididymis. But the cure of the stricture, or of the urethral affection, will certainly not cure this, and no good surgeon now-a-days believes that it will.

*Neuralgia of the Testis, or Irritable Testis.*

This, like facial neuralgia, is an extremely rare and a very intractable malady. Castration has been performed for it sometimes with success, more frequently without it. It were well in this, as in tic douloureux, if we knew with any certainty the causes which render an operation more successful in one case than another, and could apply that knowledge to the diagnosis. Unfortunately it is not so, and we are driven to the enumeration and observation of facts. Mr. Russell has known of only three cases in Edinburgh, and of these two ended favourably. They are highly interesting.

"I was once concerned in a case of this description in the person of a medical practitioner, who came to town from a distance, with the view of submitting to the removal of his testicles. After his arrival, he was put under a course of medicines, which was not of the smallest service; and as his medical friends had no farther measures to recommend, and as his sufferings were intolerable, he finally resolved on the operation. Accordingly it was performed with the most fortunate success, as he was instantly relieved from pain, which never aftewards troubled him. He recovered his health, strength, and spirits, which had been impaired by the severity and continuance of his complaint. A practitioner remarkable for the soundness of his judgment, encouraged by the success of the preceding case, adopted a similar practice in a like case, which, however, was not followed by an equally favourable result, as the patient experienced but imperfect relief in the first instance, while the complaint returned, gradually increasing in severity, till at last it attained its original violence. The next case that occurred here was treated upon other principles. The practitioner, one of the most eminent in town, perhaps influenced in his judgment by the unfortunate issue of the last case, advised the patient to submit to his sufferings with patience, in hopes that time would at last accomplish a cure. The patient followed this advice, and was relieved from his misery in the course of eighteen months."

Each case presenting different circumstances we are unable to draw any conclusion from the results, save that the two patients who recovered were in luck. In a case, but it is not a very severe one, at present under our care, connubial intercourse has been productive of some benefit.

*Gout and Rheumatism of the Testis.*

Cases of rheumatic testis have been recorded, and when we consider the fibrous structure of the epididymis and tunica albuginea, we need not feel surprised at rheumatic inflammation attacking the organ. Alix relates a case of severe affection of both testicles, which he treated ineffectually as simple inflammation, but hearing that the patient was much exposed to vicissitudes of temperature, he treated it as rheumatic, and with success. Other cases have been related in which swelling and pain of the testes were relieved by the joints becoming affected, and general rheumatic fever being established, or in which the testicles were vicariously attacked.

"The testicle is likewise liable to be affected by gout very much in the way it is affected by rheumatism. From a characteristic difference, however, between the two diseases, the origin of the attack is often more completely concealed, so that nothing appears to excite suspicion till the supervention of gout in other parts of the body relieves the testicles, and develops the mysterious nature of the case. In some cases of this kind, the attack has commenced with a sudden swelling of the testicle, which was remarkable for its extreme hardness and weight. These symptoms being by some surgeons regarded as pathognomonic of a cancerous scirrhus, have led to the unfortunate practice of removing the testicle by castration. I have seldom in the course of my own experience had occasion to witness an attack of gout in the testicle, but the attacks which I have witnessed were marked with the impenetrable hardness, and increase of weight, so characteristic of the complaint. A gouty affection of the testicle is rather a rare occurrence, so that there is not any appropriate treatment established for the cure.

Obscure as is the nature of the connexion betwen gout or rheumatism and affections of the testicle, the dependence of an affection of the testicles upon that unknown state of the atmosphere which produces an epidemic disease is still more obscure. Mr. Weddows gives a distinct history of an epidemic swelling of the testicles which occurred in the neighbourhood of Wallingford. At least, the number of individuals affected in a short space of time was so great, as to render it reasonable to ascribe the coincidence to the effect of some common cause. The disease was not dangerous, all the patients recovering under the usual method of cure."

*Varicocele.*

Mr. Russell enumerates the operations that have been resorted to for this troublesome affection—ligature of the vein or veins—division—the application of caustic over the trunk, to procure its obliteration—excision—ligature of the spermatic arteries—and castration. The mere fact of so many operations having been resorted to proves that none are safe and effectual. Mr. Russell has omitted M. Delpech's operation, exposure of the vein and obliteration of it, by the retention of a foreign substance in contact with it. We have another operation to mention, which has been employed two or three times by Mr. Briggs, the senior surgeon of the London Lock Hospital. It is the removal of a large piece of the scrotum. We cannot say how far this has succeeded, but really it appears to us to be founded on a more rational principle than any of the other methods, and to be less liable to dangerous consequences.

We have picked out some portions from the work before us, as either presenting a view or a fact that may be useful. It is rather a rambling production and might have been condensed and methodized with advantage. We fear that Sir Astley has left no room at present for a book upon the testicles.

---

### XIII. Gastritis and Dyspepsia.

Dr. Stokes has been delivering a clinical lecture on these subjects, which we will take the liberty of noticing. The lecture in question is reported in our cotemporary, the London Medical and Surgical Journal, for the 25th of May of the present year.

Dr. Stokes observes that much ignorance prevails on the subject of gastritis and dyspepsia, among British practitioners. We fear that this censure is but too well merited. It appears that the potato diet which some travellers in Ireland are much disposed to laud, is very apt to produce gastritis, which according to Dr. Stokes is extremely prevalent among the Irish peasantry. The case which has chiefly given rise to Dr. Stokes' remarks is the following.

"The patient, who is the subject of the case before us, is somewhat reduced in flesh and of a sallow complexion. He has complained of pain in the region of the stomach, extending to the back, right hypochondrium, and shoulder. He has had tenderness over the epigastrium, loss of appetite, pain and sense of distension, increased after eating; vomiting of yellow matter occurs two or three hours after taking food, succeeded by thirst. His pulse is soft and slow; tongue clear; bowels open. His illness (and this, I think, is a point worthy of remark) commenced four years ago with pain in the stomach, increased by eating and relieved by vomiting; and some time after this the vomiting began to be succeeded by thirst. The vomiting generally came on in an hour or two after taking his meals, and he threw up a quantity of yellow slime. Another important point is connected with the treatment he has undergone for this disease. He has been relieved by antiphlogistic treatment, locally employed; we treated him since his admission by leeching, blisters, and cupping over the stomach, and latterly he has been using narcotics."

The treatment which has been adopted by the Doctor has been, regulation of the diet—cupping and leeching the epigastrium—and counter-irritation. Dr. S. observes that in similar cases he has employed nearly similar treatment, that in most instances this has been followed by permanent relief, but that, where it has not been so, recourse has been had to narcotics, and particularly the acetate of morphia. Dr. Stokes is convinced of the value of narcotics in these cases. Dr. S. relates another case of chronic gastritis, which is short, and which we extract.

"There is, gentlemen, another case,—that of the patient Denham, who has been complaining of pain and tenderness in the epigastrium, with loss of appetite, and intolerable thirst. His face and extremities are œdematous, urine not albuminous, bowels confined. His tongue is red, and thickly coated with fur; his illness commenced two months since. I looked on this as a case of chronic gastritis; for, you observe, he had all the symptoms, pain, tenderness of the epigastrium, red tongue, impaired appetite, and an insatiable desire of cold drinks. We treated him by leeching and blistering the epigastrium; we gave no purgative by the mouth, but obviated the costiveness by enemata. By this treatment much good has been effected. Since the leeching and blistering his thirst, which was so excessive that I thought at one time he had diabetes, has completely declined; his tongue is much improved, and he no longer complains of any gastric pain. His appetite, however, continues bad; and it will remain to be seen by the progress of the case whether this depends on want of tone in the stomach or actual disease."

The chief reason for which we notice these cases, is, that we may advert to some doctrines laid down by Dr. Stokes on the subject of dyspepsia. He maintains that dyspepsia is a term given to disordered functions of the stomach unattended with organic lesion; or "alteration of circulation." With all deference to the authority of Dr. Stokes, we cannot assent to this position. Dyspepsia is the representative term of a certain assemblage of symptoms, without reference to their cause; it implies a certain alteration of function in the stomach, whatever may be its actual condition. The term dyspepsia in this respect resembles the term dyspnœa, or the term fever. None of them imply any definite organic change, because they may be dependent on many organic changes, or on merely functional disturbance.

It may be said, that if dyspepsia is thus a symptom or assemblage of symptoms dependent on several organic lesions, we should discard the term, and adopt one referable to the special lesion. But, in point of fact, we do this when the lesion is prominent and distinct. If there be loss of appetite, disordered bowels, malaise, depression of spirits, uneasiness in the stomach after taking meals, and slight tenderness at the epigastrium, we name this dyspepsia, not because there is no organic change, not because there is not slight inflammation of the gastric mucous membrane, not because it is purely the nervous system of the stomach that is at fault, but because the actual lesion is not certain, and it is, therefore, more convenient to erect the assemblage of symptoms into a disease. If those symptoms increased in severity, if vomiting supervened, with more distinct fever and more decided pain and inconvenience in the region of the stomach, we should then discard the unphilosophical term of indigestion, and adopt that of the evident lesion—gastritis. It is true that it is somewhat unphilosophical to erect an assemblage of symptoms into an entity, and term it one disease; but it would be still more unphilosophical to assign to those symptoms one definite cause, when facts do not confirm it. It is the lesser evil that we choose—it is in some degree an acknowledgment of ignorance; and, in proportion as our certain knowledge advances, will such exhibitions of imperfect science disappear.

We indulge in these remarks, because we are astonished that a man of such information and such talent as Dr. Stokes should have fallen into an error so vulgar and so palpable, and because it is a practical question. If we believe, and especially if we do so independently of strict induction, that dyspepsia depends on one cause, and still more if we believe, with Dr. Stokes, that that cause is affection of the nervous system, what can we do but adopt an empiric mode of treatment? But, on the other hand, if we look on the malady as one of function, dependent on several conditions of the organic apparatus, we are necessarily forced to investigate the particular case, to see on what the dyspepsia depends in the individual. In one, we find comparatively little epigastric tenderness, the circulating system is below par, there appears to be no local vascular action, and such a person bears tonics and generous diet —in another, there is an epigastric tenderness, and a foul or red tongue, and an irritable stomach; he is benefited by very mild aperients, with sedatives, counter-irritation to the epigastrium, the mildest farinaceous food, and the careful abstinence from stimulants or tonics, until all tenderness and all vascular irritation is removed: in short, we might enumerate several shades of difference, and the judicious practitioner is he who is aware of the fact, and shapes his treatment accordingly.

It was, strange to say, an ignorance of this fact, and a consequent bad system of treatment, that gave Mr. Abernethy his reputation in the management of dyspepsia. He wrote at a time when men were merely breaking ground in true pathology—when systems had still their hold, and names their charms. He assumed, but he assumed boldly; and, with much sterling merit and some meretricious influence to support him, he persuaded the world that purging, that blue-pill and black-draught would agree with their stomachs. We wish those stomachs joy of their contents.

Dr. Stokes makes several judicious reflections on chronic gastritis; among others, that it is impossible to lay down, or, indeed, in practice to discover, the difference between chronic gastritis and dyspepsia. This is not to be wondered at, since, in many instances, chronic gastritis is the essential cause of dyspepsia. The following remark is founded on the misapprehension of the real value and import of the term to which we have already alluded. "I feel convinced (says he) that chronic gastritis is very often confounded with dyspepsia by British practitioners. It is treated as disease of the liver, by blue-pill and black-draught; it is treated as dyspepsia, by tonics and stimulants; it is treated as constipation, by drastic purgatives." That chronic gastritis is frequently overlooked by practioners is, we fear, too true, and we fear that their treatment of dyspepsia is not always so judicious as might be wished. The purgation system has taken too deep root to be readily destroyed. But we protest against the inference that may be fairly drawn from the sentence which we have quoted—that dyspepsia is, as a matter of course, to be treated by tonics and stimulants.

Dr. Stokes mentions one or two other facts, of so interesting a character, that we cannot close this short article without extracting them.

"There is another case which I wish to notice: it has been, I believe, one of an acute character; I allude to that of the man in the Fever Ward. This person, after committing an excess in drinking, got sickness of stomach and vomiting. In your investigations of any case which comes before you, it is of importance, towards forming a correct diagnosis, to hold these two things in view,—the exciting cause, and the first symptom of disease. Here you have, in the first place, excitement of the stomach from the use of spirits, and afterwards irritation, manifested by the vomiting. This was followed by loss of appetite, constipation, pain in the lower part of the left hypochondrium, foul tongue, red at the tip, symptoms which indicate irritation of the mucous membrane of the stomach and intestines. When he was admitted into the hospital, however, what he chiefly complained of, and what were certainly the most prominent symptoms, were tightness across the chest, great difficulty of breathing, and harassing cough. His cough was indeed very severe, his sputa slightly tinged with blood, his breathing very much accelerated, and, to a superficial observer, he would appear to labour under chest-disease. But, remark, we found out that he had been complaining of these symptoms for about three weeks, and consequently, if they had been pulmonary symptoms, they must have proceeded to a very alarming extent in that time. Mr. Lees examined him by percussion and with the stethoscope, but could not detect any disease of the lungs, and he was examined by myself on the next day with the same result. Moreover, the patient had been previously treated for pulmonary disease without success. We were, therefore, led to conclude that there was no original disease of the lung, but only sympathetic irritation, depending on gastritis. We took small quantities of blood from his arm, leeched the epigastrium, kept his bowels open by enemata, and under this treatment we saw all his symptoms disappear, as it were, by magic."

Dr. Stokes mentions two other cases. A man was in the hospital, labouring under "what might be called a tussis firma." He was treated with leeches to the epigastrium and iced water, with most signal benefit. He committed some excess in eating, and had a return of his complaint; he was treated again in the same way, and recovered. Dr. Stokes attended a lady some years ago, who complained of some feverishness, with very severe and harassing cough. He treated it as a case of fever, with irritation of the bronchi. The fever declined, but the cough continued, and Dr. S. was embarrassed. The lady remarked one day that she had been under Dr. Cheyne's care for a similar complaint, and had derived much benefit from leeches to the epigastrium. On this hint Dr. S. acted; the leeches were applied immediately, and the patient's cough entirely disappeared.

These cases are certainly in favour of the belief in "a stomach-cough." But we fear the sin is on this side, and that real pulmonic disease is too often ascribed by practitioners to dyspepsia, or affection of the liver. The Editor of this Journal has seen several melancholy instances of the injurious consequences of this popular error. The opposite mistake is, at all events, the safer.

Before we conclude, we will make one remark. It gives us the highest pleasure to see such eminent physicians and surgeons of Dublin as Dr. Stokes, Dr. Graves, Mr. Crampton, and others, publishing essays and clinical observations in the journals. These gentlemen appreciate rightly the times and the public mind, and it is most gratifying to us to perceive, that they have not only the power, but the inclination, to diffuse information and advance the profession. We bid them persevere in the good cause.

---

### XIV. MR. ROBERTSON ON DRY CUPPING.*

Mr. Robertson, an intelligent surgeon residing in High Holborn, has published some interesting cases of pain de-

* Lancet, Jan. 19th, 1833.

pending on various causes relieved by dry cupping. The cases in which he would recommend it are those in which the pain is dull though severe, deep-seated, chronic, not much increased by pressure, or has refused to yield to ordinary means. The way in which he generally employs the remedy is to throw a very minute bit of paper touched with ether or turpentine, lighted, into a large glass or tumbler, and press it down in the usual way. This is a very effectual and a very convenient method of obtaining the requisite exhaustion, and is attainable when the regular cupping apparatus is not. We will select two cases out of eight related by Mr. Robertson. They are the most interesting and perhaps the most satisfactory.

Case 1. *Spasmodic Pain in the Loins, post Coitum.*

"A stout young gentlemen, and in fine health, was seized shortly after connexion, with most violent deep-seated pain in the region of the *left* kidney; so severe that he was unable to walk, stand, or sit. Lying on his back on bed, or on a sofa, gave a little, and only a little, relief, and frequently did not relieve him at all. He had been often attacked similarly before, and traced it distinctly to connexion. The peculiar sensation in the part commenced immediately after coitus, and could be felt distinctly increasing more and more till it ended in a paroxysm. Sometimes the sensation went off altogether, particularly if he carefully kept the recumbent position; but when it did not, four or five hours would intervene before the paroxysm came to its height. In one instance he was attacked severely while walking home, and had very nearly fallen down in the street. In another he was awoke in the night. Twenty or thirty leeches relieved it the first time. In about two months it came on again, when bleeding at the arm, and 70 leeches over the seat of pain, gave him only moderate ease.

After some months' interval it returned a third time, and leeching, even to a very great extent, seemed to have altogether lost its power. A blister and some internal medicines were prescribed by a physician with little relief, when I ordered 20 leeches to the anus; this, and the recumbent position for two days, subdued it.

A fourth time he felt it coming on. This was at midnight (connexion having taken place some four hours previously), and when I saw him, the pain in the left lumbar region seemed frightful, deep-seated, and of that peculiar nature, that he could bear almost any pressure on the part without shrinking. He lay in bed writhing like a serpent.

I felt loath to bleed him to such an extent as had been requisite formerly to subdue it. Leeches were not at this time conveniently to be had, and I determined to try the effect of dry cupping. A very large tumbler was put over the part, kept on for a minute or two, till it seemed to gall him; taken off, and replaced three or four times. The effect delighted and almost astonished me. One minute after it went on, the most perfect relief was felt; the pain was entirely gone; so afraid was he of its return, and so keen to have the glass on, that he insisted upon having it on and on, till the edges of the tumbler had almost cut into the muscles! This he declared he cared not for. It was a trivial thing compared with the dreadful and insufferable pain in his side. I knew him for years afterwards, and though the cause was continued as before, he never had any return."

Mr. Robertson supposes, reasonably enough, that the pain in this instance depended on spasm of one of the lumbar muscles.

Case 2. *Pain in the Left Umbilical Region.*

"A very interesting young lady, the wife of a friend of my own, left —— for Edinburgh, immediately after the marriage ceremony was performed; and when about half-way, was seized, while in the carriage, with most violent pain in the left umbilical region. Her husband managed to get her by easy journies to Edinburgh, where she remained about three weeks, and was bled, blistered, purged, and put through all the ramifications of the strictest antiphlogistic system.

At length she was obliged to be brought home by short stages, a distance of 40 or 50 miles; and was ultimately relieved by turpentine enemata, which brought away some discoloured hardened feces. Her menstrual periods had for years been attended with extreme pain. During the two years which followed marriage, she was said to have had two miscarriages. During the third she miscarried again, and was getting round, when I was suddenly sent for, on account of an alarming pain *in the very spot which had formerly been so productive of suffering*. She could not account for it; she had been lying quietly in bed, and had been eating and drinking nothing to produce it. The lochial discharge went on as usual, and her bowels were natural, as she had, ever since her former attack, used Maw's instrument. Leeching was proposed, but to this I objected, on account of the loss of blood she had sustained so lately, and from the effects of which she had not as yet recovered.

I determined first to try dry cupping; she assented, and as the things are always at hand, in five minutes she was so well as to be able to joke with her husband and me about 'the very troublesome wife' the former had. It never returned.'

---

### XV. THE MEDICAL BOARDS.

Our Medical Boards are, we fear, in a bad way. The filthy fingers of reform that have polluted so many establishments remarkable for their antiquity, and consecrated by memorials of the wisdom of our ancestors, have begun to touch the outworks of our own popular institutions. Those whose duty and whose interest it is to cling to those institutions to the last, have but too much reason to consider this the prelude of a general attack on the strong holds themselves. The work of spoliation which is busy with all that is dear to them, which is now seen encroaching on the hallowed prerogatives of law, and now on the sacred establishments of church, is too likely to be enacted on the sister frame-work of physic.

The present may indeed be considered as the age of bold bad men. Those who have long been connected with our medical institutions, who have thrived and lived by them, and are consequently best acquainted with them, who unite with a keen perception of their beauties a necessary knowledge of their defects: these gentlemen, we say, aware of the difficulty of amendment, of the weakness of human reason, and the fallacy of theory, have declined to enter on the hazardous experiment of reform, have been prudently satisfied to let well alone, and have justly considered that at present there is a certain benefit to a few, whilst popular alterations would disperse and subdivide it among the many, so that it would not afford substantial livelihood to any. If reform come, they wash their hands of it, and when the mass of the profession, the ignavum pecus, have completed the work of destruction, have levelled the gilded roof and the ornamented column, they at least will have been guiltless of the Vandalism.

The deplorable state of feeling which exists at present, is depicted in characters too legible, in the destruction of the National Vaccine Institution. It is true that many ignorant and more evil-minded individuals had looked upon this as a job, and it is true also that the Government had unhappily been deluded into a similar belief. It is the prevalence of such a disposition in governors and governed, that is calculated to excite a feeling of dismay in all who wish well to medical science. The following, and unhappily the last report from the National Vaccine Institution, is a sufficient answer to its calumniators. It is at all events a splendid thing in the annals of the institution that it has died in the arms of glory; happy if, like Decius, its destruction might appease the infernal gods, and avert their wrath from the remaining pillars of the state.

"TO THE RIGHT HON. LORD VISCOUNT MELBOURNE,
*Principal Secretary of State for the Home Department.*

---

*National Vaccine Establishment,*
*Russel-place,* 21*st Jan.* 1833.

My Lord,—The Board of the National Vaccine Establishment has executed the benevolent purposes of Parliament this last year with its usual zeal, and with all possible success.

The number of Persons vaccinated in the metropolis and suburbs by its own immediate agents, within the last twelve months, has exceeded that of any former year by 3000, and the means of giving the protective process have been distributed by us to more than 100,000 others in various parts of the world. To maintain such a supply of the vaccine lymph, and to be prepared to answer on the instant the incessant demands which are made upon us for it, nothing less than a National Establishment is adequate; and accordingly we have found that where the charity of individuals, however abundant and well organized, has been appropriated to institutions having the same objects in view in the country, such institutions have always failed.

The opportunity of taking the lymph from a vesicle in progress, in order to be most successful, should be taken between the seventh, and eighth days, which is so limited a period, that, unless there be a large number of vaccinators to contribute continually their respective quotas of authentic lymph into a common depôt, there is danger of the store failing when it is most urgently wanted.

The small-pox has been prevailing with its usual fatal results in various parts of the country since our last report; and magistrates frequently write to us to express their regret that they cannot prevent ignorant persons from going about the country to inoculate; but we still live in hopes that the good sense of the people will discover the superior advantages of vaccination, when it is repeatedly stated to them as a fact, that, of an equal number of persons vaccinated and inoculated, only so many of the former will be capable of taking the small-pox afterwards, and that in a safe degree of the disease, as are found to die by the latter.

(Signed) Henry Halford,
*President of the Royal College of Physicians.*
Thomas Hume, M.D.
*Censor.*
John Painter Vincent,
*President of the Royal College of Surgeons.*
Clement Hue, M.D.
*Registrar.*"

Our contemporary, the London Medical and Surgical Journal, which appears to rejoice at the abolition of the Board, mentions a few circumstances connected with other boards. It observes that, among the expenses of the colleges of Scotland, a return of which has just been made to Parliament, two items of a curious description are to be found—a salary to His Majesty's limner, and one to His Majesty's historiographer. We have no doubt that these gentlemen render important services to medicine—if they could be known. There are two other items, which probably can be equally satisfactorily explained.

| | |
|---|---|
| To His Majesty's Plate at the Races.. | £100 |
| To the Caledonian Club .......... | 100 |

It might seem absurd to a superficial examiner, that horse-racing and medical appointments should be united. But when rightly viewed, there is considerable analogy and connexion between them. One is a race of the bodily, the other of the intellectual being. There is equal discipline beforehand, currying, combing, trotting, and gingering—equally hard work in the race—and, in both cases, a good stall and good provender afterwards.

---

### XVI. The Factories' Regulation Bill.

We are forcibly struck with the unpretending and philosophical manner in which our contemporary, the Phrenological Journal advocates the extension of education and the moral advancement of the community. There are some observations on the proposed restriction of the labours of factory children to ten hours daily, which do credit to the judgment and the feelings of the writer. At the present period, when a commission, partly consisting of medical men, is visiting the manufactories, it may not be amiss to offer a few remarks upon the subject. It is one in which medical observation must and should form the basis of legislative enactments, for were not the health and existence of the children at stake, there could be no valid reason why Government should interfere between capital and labour. There are some, we believe, among others, Mr. Hume, who conceive that the Government should not interfere even in such a question as this. We must confess that we differ from these gentlemen, and we think they are yielding overmuch to system and to abstract principles, when, for their sake, for the sake, in short, of free competition and free trade, they sacrifice the health, the comfort, and the lives of millions, and those the most defenceless. Mr. Cobbett observed, and very rightly, that the parties at fault were not the manufacturers. The children are not slaves; they are not compelled by the masters to enter into the factories. No; it is the distress or the avarice of the parent that sends his child to the mill, and Parliament does in reality protect the offspring from the cruelty, perhaps in general unavoidable, of the father and the mother.

Our contemporary justly observes that the question, stripped of all factitious considerations, is simply this:—Can children support twelve hours in the twenty-four of labour? We answer unhesitatingly that they cannot; and were the members of the medical profession, from John o'Groat's house to the Land's End, polled on this particular point, we know that, almost to a man, they would make this answer. There need neither commissions nor commissioners to tell us this—our common sense and common observation are sufficient to decide the matter. The children of the middle and opulent classes in great towns, who have no such appalling amount of toil, who do not breathe the heated atmosphere of the mill, who are well clothed and well fed, and comparatively well exercised, are found on the whole to pine, and to require the pure air of the country or the bracing atmosphere of the coast. And we are told that the child of the Manchester mechanic, labouring for twelve hours in a manufactory, heated to 70 or 90 degrees, with an atmosphere smelling of oil, or turbid with the fine dust engendered in the processes—obtaining only a hurried or a scanty meal—ill clad, and passing from such an atmosphere to the open air, and probably to a wretched tenement—fatigued at a monotonous employment, amidst the continual whizzing of machinery—its spirit cowed with the dread of punishment from the hands of the overlooker, or dismissal, and still worse treatment at home—we are told that children can bear all this, and that they are not *very* badly off. Let those who choose be satisfied with the excuse; but sure we are, that our profession, best acquainted, as it must be, with what human nature can and cannot bear, will repudiate it without a moment's hesitation.

Were a Malthusian to argue in support of twelve hours of labour, we might acknowledge his consistency, however we disapproved of his inhumanity. But the Malthusians are silent, and whatever they may think, they have not ventured to defend the present abominable system. If, indeed, their doctrine be correct, and if the better feelings of the age continue to diminish the unwholesomeness of the employments of large bodies of the community, we do an ultimate evil at the expense of both manufacturers and children.

Dropping, however, these considerations, the question has formally and solemnly been put to us, the medical profession, and it has been put to us by the public as well as by the Parliament, do we or do we not think twelve hours of labour consistent with the health, a merely tolerable degree of health, of factory children. The profession, so far as it has had an opportunity of speaking out, has replied that it cannot be. We trust that the medical men on the commission, able and upright as we know that they are, will not allow themselves to be seduced by persuasion, by the indifferent tone of the society of masters into which they must be thrown, or by any considerations of what may be palatable to parties or to individuals, but will do their duty fearlessly, and respond to the general sentiment of the profession. The eyes of that profession are on them, and we do not doubt that they will prove themselves its representatives.

We have lost sight of our contemporary, to whom we referred at the commencement of these remarks. He observes that whether we look at the effects of the present amount of factory-labour on the muscles and bones—the brain and nervous system—the digestive organs—or the organs of respiration, we shall find them alike condemnatory.

It is proved by evidence that deformity prevails among the children, that their growth is retarded on an average nearly two years, that few if any attain the usual degree of robust development or vigour, and that many become thin, feeble, and wretched from the day they enter the mill. The witnesses are unanimous in their complaints of the dulness, heaviness, and sleepiness of the children, in the impossibility of keeping them awake at school, or exciting them to further labour, after their labour is done, whilst in many mills corporal punishment is or was regularly resorted to in the latter part of the day, to produce wakefulness, and at this time, too, the accidents from entanglement in machinery usually happen.—The children are subject to convulsions, typhus and other diseases of exhaustion.

Want of appetite, bad digestion, and sallow unhealthiness of aspect are prevalent among the children. In many of the factories no pause is allowed for meals, and the unhappy wretches are obliged to eat in mouthfuls, as they can best snatch a moment for the purpose, while their food, from the lowness of the wages, is at best insufficient to maintain them in vigour, and is often spoiled by the floating dust. Few too escape pulmonary disease either in early, or, if happily they attain it, in advanced life.

The temperature of the factories in many stages of the operations, requires to be kept as high as from 70° to 90°, the average of tropical climates.* Some of the masters have invited an inspection of their mills, and of the children working in them, But our contemporary justly observes that this is the best part of the case. We see at the mills only those who *can* work, we do not see those who are disabled or diseased and *cannot*. Inspect the mills, but inspect also the houses of those who are and who have been in the factories—do justice to the master, but do justice also to the child—in fact, view the whole, and not merely the most or the least favorable part.

We need scarcely say that there are moral and political considerations, as well as medical, at stake. Our immense manufacturing population is becoming physically degenerate and degraded, the decrepid and deformed begetting their like. They are growing also more vicious and more turbulent, and all agree in the existence of a spirit in the manufacturing districts which cannot be regarded without the deepest alarm. Lord Brougham has stated the state of education in this class is deplorable almost beyond relief. And where is the wonder of all this? Underfed, overworked, without a prospect of bettering his condition, or passing from what is after all worse than slavery, the artisan has no tie to bind him to his country or her institutions, and in every master he sees a sordid and unfeeling tyrant, in every man better off than himself a spoliator or a speculator in his sweat and toil. Government is in his eyes a combination against him, and the whole nation resolves itself into two great classes, the oppressors and the oppressed.

---

XVII. The Heliotrope; or Pilgrim in Pursuit of Health. 8vo. 1833.

Whether the Pilgrim be a physician or

---

* This, by recent confessions, is an exaggeration.—Ed.

patient, certain it is that he is a poet—and one of some promise. The poem itself will be interesting not only to those who seek health from Italian skies, but to those who delight in historical recollections, and beautiful description of celebrated scenes. The author writes a good deal in the style of Byron, and gives a very animated delineation, not only of the voyage to Italy, but of his journies in search of health subsequently. We greatly fear that the youthful poet has over-rated the salubrity as well as the pleasure to be derived from the climate of fair Italy. The following stanza on the bay of Naples will exemplify this remark, and at the same time, convey some idea of the style of the author.

——— Here if thou lovest a clime
Where health may flourish--rankling care decrease
And beauteous Nature smooth thy stream of time—
Here, in Campania's *Aprosapolis*—
Repose! and feast thy soul with scene sublime—
* * * * *
The sunbeam shall not smite thee, for the sea
Tempers its fervour; Winter's kindly ray
Shall never chill thee, for the myrtle-tree,
Pomegranate, palm, and citron, shade the bay
With fruit and foliage; Nature's face shall be
Thy book and mirror—one long Summer day
Thy life; and when at last thou takest thy rest,
Unfading Spring shall fold thee in her breast.

If the physician could corroborate this description of the poet, then indeed would Italy be a paradise as well as a *portus salutis!* But it unfortunately happens that the ratio of mortality, even in this delightful Parthenope, is full double that of our own foggy, stormy, and rainy isle!

---

### XVIII. MARCH OF INTELLECT.

Within the last few weeks medical science has made some most rapid strides. In Paris a female underwent a formidable surgical operation while in a state of somnambulism, produced by animal magnetism. M. Chapelain operated on the senses—M. Cloquet with the knife. The somnambulic sleep, or insensibility, lasted 48 hours, and on awaking, she experienced some emotion at the sight of her children, and on learning that the operation had been performed. The magnetiser checked this emotion by immediately throwing her into a sleep again. We hope that the æra of suffering under the knife is over. No doubt that death itself will soon be divested of his sting!

At home, a most important discovery has been made—the attraction, or rather the extraction of tic douloureux by the north pole of the common magnet. A man in St. Thomas's Hospital had long suffered from neuralgia of the finger, and all the remedial agents—including iron, colchicum, and the lobelia inflata, had failed. The instrument is of the horse-shoe form, ten inches in its longest axis, and five in shortest. It is composed of five layers of metal—the central being the longest, and the whole bound with strong riband. The North pole of the magnet was gently passed five or six times down the sides and back of the affected finger, and then rested on the central joint. The result was such a cessation of pain that the man could "knash his fingers into the palm of the hand with ease and comfort." Dr. Blundell, the operator, shewed that the power of the instrument did not cease here. He reproduced the pain in a most intense degree by applying the South pole of the magnet. After permitting the tic to continue for a short time the North pole was re-applied, and the pain again removed. A similar operation was equally successful in two other cases!

These are extraordinary facts; but those who have witnessed the capricious assaults and retreats of that merciless tormentor, tic douloureux, will have some doubt as to the future success of the magnet, when the novelty of the remedy has passed over. Still, as there is no juggle in the business, as is evidently the case with "animal magnetism," we recommend a full trial of the operation.*

A proposal has been made by Mr. Lucas, of Weston Street, to dissolve the calculus in the bladder, by means of "solvents introduced into a sac in

---

* Since the above was written, we learn that the operator gives each patient half-a-crown! So, then, there is a silver magnet to assist in extracting the TIC.

which it (the calculus) has been isolated." As Mr. Lucas has not given us any description of the instrument, though he affirms that it is perfectly free from danger, and applicable to all cases, we must wait for a further development of the plan.

---

### XIX. Labour or Ennui. Which is the greater Evil?

The whole nation, from Shakespeare's cliffs to John o'Groat's, is labouring under a severe attack of *acute* humanity —and the medical profession has caught the contagion;—the moral influenza being decidedly infectious. Sir David Barry, lucky chap! has been packed off to the Highlands, for change of scene from St. Petersburgh, and is now (or has been lately) dancing strathspeys in Perthshire with his brother commissioners. We shall soon have a volume of reports on the nature of the new epidemic, its prevention, and cure.

It has been discovered that a portion —a very small one indeed—of society is oppressed with too much labour, and the sympathies of the whole population are called forth on this interesting occasion. There is an evil, however, in existence, infinitely greater in itself, and far more injurious to society, which seems to have escaped the notice of our brethren in particular, as well as of the public at large, viz. the WANT OF LABOUR. The miseries which spring from this source are incalculable! IDLENESS, both voluntary and involuntary, is the parent of crime and the inexhaustible fountain of discontent. God forbid that we should advocate cruelty, drudgery, or slavery, at home or abroad; but we cannot help thinking that the outbreak of outrageous sympathy for a few thousand boys and girls, who are no doubt overworked, is quite disproportioned to the cause. Let the philosopher and philanthropist look around, and contemplate the prodigious mass of misery and crime produced by the want of employment—and let them devise schemes for alleviating the evil. Every political economist, from Malthus to Martineau, acknowledges the unhappy consequences of a redundant population—an evil constantly and rapidly increasing! Plagues, pestilences, famine, sickness and premature deaths, are the means by which nature and art are endeavouring to lessen the magnitude and growth of the evil. The "FACTORY LABOUR" may be, and probably is, one of the means inseparable from a high state of civilization and mechanical improvements, which abridge the duration of life and check redundant population. It may be abolished; but the same consequences will spring up under some other head. The swarms engendered in our manufacturing towns will die prematurely, whether by excess of labour or defect of food; and the problem has yet to be solved, which is the lesser evil. We have visited many of these factories, and unless our observations are inaccurate or our perceptions obtuse, we have come to the conclusion, that the destructive influence of excessive work is monstrously exaggerated. In several localities—we shall only particularize Lanark—the operatives appeared remarkably healthy—and, in most of the others, we very much doubt whether the mere amount of labour conduces so materially to the abridgment of existence as is represented. Where can we turn our eyes without seeing the destructive operation of causes connected with the actual state of society? Are our nobility exempt from them? No, indeed! the ennui of civilized life, *the want of work*, sends five hundred to a premature grave, for every unit of mortality produced by "*factory labour*," or by excess of muscular exertion any where. Go, for instance, into that splendid REFECTORY (Verry's) in Regent-street, and look at that beautiful piece of wax-work on your left, who sits smiling on the whiskered ice-eaters in the saloon. Her flesh is like double-refined spermaceti—and her blood like water, with a slight dash of port-wine. Her span of existence will be very short. And so of ten thousand other maidens in this metropolis, whose sedentary avocations push them prematurely into the silent tomb! Why not introduce a bill for the amelioration of this immense class? Is it not an abominable shame, that so many fine young men should perish by the sword, by disease, and by "over-exertion" during war? Should there not be an act of parliament to prevent shipwreck and scurvy among our valiant tars? Why are men allowed to go to the East and West Indies to die of hepatitis, dysentery, and yellow fever, long before they have attained the honorable age of three score years and ten? Why are coal-heavers allowed to drink so much "*heavy wet*," ending in dropsy? Why are chimney-sweepers *forced* to live upon soot, and die of cancer of the scrotum? Is it not inhuman to permit so many thousand men to labour in the bowels of the earth, deprived of light and air, save when the fatal choak-damp explodes around them? Are not our delicate female children *forced to labour* at music, drawing, and Italian, to the great detriment of their health, when they ought to be out running among the fields and hills? Finally, (and this is an argumentum ad hominem,) is it not grievous to think that a large class of medical men themselves are often *forced* to continue at their *labours*, not for ten or fourteen hours merely, but frequently for 24 or 36 hours, without intermission! Why does not the faculty, and especially the large obstetric class, apply to the Legislature for an act that may limit all *labours*, whether natural, complex, or difficult, to ten hours' duration?

To conclude, we do not object to the regulation of labour in factories; but we would take a wide and comprehensive view of things, and sweep away all other grievances, many of which are infinitely more productive of sickness, death, and misery, than the fourteen hours work in cotton mills.

---

### XX. Dr. Carswell on Carcinoma.

In the Review Department, page 144, we alluded to Dr. Carswell's second fasciculus of Illustrations of the Elementary Forms of Disease, but we had not space to notice at large the opinions and statements of the able author. We shall do so here.

The subject of the present fasciculus is carcinoma, which Dr. Carswell considers under the general head of heterologous formations. Their essential character depends on the presence of a solid or fluid substance different from any of the solids or fluids which enter into the healthy composition of the body. But this substance presents such varieties as to constitute diseases of different kinds, which have been named scirrhus, fungus hæmatodes, calculus, &c. Dr. Carswell includes under the following denominations what he conceives to be distinct diseases belonging to the class of heterologous formations, viz. carcinoma, melanoma, pyonoma, tyroma, lithoma.

Carcinoma, then, is a genus in which Dr. C. comprehends as species, scirrhus; common vascular or organized sarcoma; pancreatic, mammary, and medullary sarcoma; and fungus hæmatodes.

"The following reasons may be assigned for thus grouping together under the generic term of Carcinoma, so many diseases generally described as differing widely from that which is commonly known by this designation. 1st. They often present, in the early periods of their formation, certain characters common to all of them, however much they may differ from each other in the subsequent periods. 2d. They all terminate in the gradual destruction or transformation of the tissues which they affect. 3d. They have all a tendency to affect several organs in the same individual. 4th. They all possess, although in various degrees, the same reproductive character."

We have often expressed our opinion of nosological classifications. If the subjects are such as to admit of a simple and natural arrangement, so much the better. But if the arrangement is at all forced, the object of a classification, facility, is lost, and nature is sacrificed to an insane love of system. We doubt whether we are justified, in the present state of our knowledge, in accepting Dr. Carswell's classification. The structure of the vascular sarcoma, or of the mammary sarcoma and of scirrhus are very similar in certain stages, but the diseases, on the whole, are exceedingly dissimilar in progress and in virulency, and we say again that we doubt the propriety of uniting both under the common appellation of cancer.

Dr. Carswell admits that there are differences of considerable importance between these diseases, but that they are referable to two states of the heterologous deposit. The first is that in which this deposit has little or no tendency to become organized; the second exhibiting a greater or less disposition to become so. He, therefore, divides carcinoma into two species, the first of which he terms scirrhoma, the second cephaloma, the heterologous deposit presenting itself in them under various forms, which may be regarded as constituting so many varieties of each species.

"*Varieties of Scirrhoma.*—The varieties of scirrhoma are determined chiefly by the relative quantity of the Heterologous Deposit, the manner in which it is distributed, and the difference of colour and consistence which it presents. Thus, it may be collected in numerous points in the form of a hard, grey, semi-transparent substance, intersected by a dull white or pale straw-coloured fibrous, or condensed cellular tissue, and, as such, is commonly denominated *Scirrhus.* When it assumes a regular lobulated arrangement, so as to represent an appearance similar to a section of the pancreas, it forms what was called by Mr. Abernethy the *Pancreatic Sarcoma.* Again, it may be disseminated uniformly throughout the texture of an organ, which it converts into a solid substance resembling a slice of raw or boiled pork, and is then called by the French the *Tissu Lardacé.* Lastly, when it presents the appearance of firm jelly, and is collected into masses of greater or less bulk in a multitude of cells, it is the *Matière Colloïd* of Laennec, the *Cancer Gélatiniforme* or *Aréolaire* of M. Cruveilhier.

*Varieties of Cephaloma.*—The principal varieties of cephaloma are derived from the appearances which the Heterologous Deposit presents either in different organs or at different stages of its development. When it presents the appearance of firm coagulable lymph, or fibrine deprived of the red colouring matter of the blood, possessing a uniform, fibriform, or lobuliform arrangement, with a certain degree of transparency and vascularity, Mr. Abernethy gave to it the name of *Common Vascular* or *Organized Sarcoma.* In this state the Heterologous Deposit is generally collected into a mass of greater or less bulk, in which few or no traces of the proper tissue of the organ in which it is contained are observable. If, on the contrary, it be uniformly disseminated throughout the texture of an organ, so as to transform it into a substance resembling a section of the mammary gland, or the udder of the cow when boiled, the appellation of *Mammary Sarcoma* was given to it by Mr. Abernethy. When it presents an appearance similar in colour and consistence to the substance of the brain, it was called *Medullary Sarcoma* by the same distinguished surgeon; *Matière Cérébriforme* or *Encephaloïde* by Laennec; *Spongoid Inflammation* by Mr. Burns. The *Milk-like* tumour of Dr. Monro; the *Soft Cancer* of various authors; and the *Pulpy* testicle of Dr. Baillie, are names which have been given to the same state.

Of all the varieties of cephaloma, the last is that in which a vascular organization is most conspicuous; and as the coats of the vessels with which it is supplied are remarkably delicate, the circulation of the blood through them is readily interrupted; hemorrhage from congestive rupture takes place, and the effused blood is mixed in greater or less quantity with the brain-like matter. From this accidental circumstance, together with the protrusion of this substance through the ulcerated integuments for example, in the form of a bleeding fungus, it has been described by Mr. Hey and also by Mr. Wardrop, under the appellation of *Fungus Hæmatodes.* Sir Astley Cooper calls it *Fungoid Disease.*"

Dr. Carswell justly remarks that although thus divided into those which are organized, and those which are not disposed to become so, it must be granted that the one sometimes passes into the other, and that we know of no characters which will enable us to say, that the incipient deposit will or will not become subsequently organized. Thus scirrhus may pass into fungus hæmatodes, and we often meet with all the varieties of both species not only in the same individual, but also in the same organ.

We think there can be no question that these malignant diseases, as they are denominated, do frequently run into each other, that they may co-exist in the same individual, and that, looking at structural characters only, they frequently invade simultaneously the same organ. But there are other points to be considered besides apparent structure, in forming an opinion of the nature of a morbid growth. If, for instance, it be found that a tumor possess a structure very similar to that of scirrhus, but that it occurs under circumstances differing from those under which that disease occurs—that it runs a different course—and that, if removed, a similar production does not appear in other organs, we may reasonably conclude that such a tumor is not scirrhous, however slight the structural distinctions may be. The case that we have put is not an imaginary one. Tumors occur in the breasts of young females, that greatly resemble scirrhus in appearance, but, when removed, leave the patient permanently well. These and other considerations prove, we think. two things; first, that mere obvious structure is not sufficient to enable us to classify tumors; and, secondly, that much is yet to be learnt respecting varieties of structure.

*Seat and Origin of Carcinoma.*—If investigated in its earliest stage, we ascertain with greater or less facility, that the carcinomatous deposition becomes evident to us, either as a product of nutrition or secretion. In the former case, it is deposited like the nutritive element of the blood, enters into the molecular structure, and assumes the form and arrangement of the tissue or organ into which it is introduced. In the latter, it makes its appearance on a free surface after the manner of natural secretions, as on serous surfaces in general. Proceeding still further, in our researches, we find this substance existing not only in the molecular structure and on the free surface of organs, but also in the blood.

Dr. Carswell proceeds to describe the formation of carcinoma in the molecular structure of organs, and for this purpose he selects the liver and stomach. If we take the liver, the first change which is perceptible is a slight change of colour, observable within a very limited and well-defined space, and distinctly seen to exist in the *acini.* This change of colour may take place in a single acinus, or in several of these bodies successively or simultaneously. The red or yellow colour which they naturally present gradually disappears, and is succeeded by a pale milk white or straw colour, accompanied by an increase of their consistence. But the most important circumstance is, that while these changes of colour and consistence are taking place, the form and bulk of the acini remain unaltered, shewing, of course, that it is a mere transformation of the tissue, through the agency of nutrition. As we trace the transformation onwards, we can perceive the altered acini forming groups, the re-union of which constitutes masses of altered structure, of greater or less dimensions.

In the stomach, this alteration is chiefly witnessed in the muscular coat, on account of the difference of colour between the muscular and cellular substance.

"The change of colour which accompanies the presence of the heterologous deposit in this tunic is hardly perceptible except in the muscular fibres. These, however, become pale, and acquire an increase of consistence; but their bulk does not appear to be increased at first, and they retain their form and distribution. Such, also, is the state of the intermuscular cellular tissue at the same period, except, as I have said, as to colour, which is not sensibly changed on account of its being naturally pale. By-and-bye both acquire a greater or less increase of bulk, become remarkably distinct, and present that fibriform arrangement, hardness, and transparency which are regarded as so characteristic of scirrhus. At a more advanced period of the disease, we no longer trace this nutritive process of transformation; the muscular and cellular tissues being converted into a homogeneous mass, which is afterwards softened down, or assumes the mammary, medullary, or hæmatoid forms of carcinoma."

Dr. Carswell next alludes to the formation of carcinoma on serous surfaces, the most simple form, perhaps, which it presents. The heterologous material is found to be effused on the free surface of the pleura and peritoneum, without their having undergone any obvious previous change whatever. Multitudes of tumours are sometimes met with on these two surfaces, varying in bulk, consistence, and colour. Some of them are as large as a plum or an orange; others of the size of cherries or peas, and composed of a substance resembling pork, the mammary gland, brain, or a mixture of the latter, fibrine, and blood. Scattered among these larger masses are a number of smaller bodies, some of them so minute as hardly to be perceived by the naked eye; others varying from the size of a pin's head to that of millet or hemp-seed. They present the same characters as the former; even the smallest of them may be nearly as firm as cartilage or as soft as brain; pale, red or bloody.

Dr. Carswell believes that, under such circumstances, the constitutional origin of the disease, that is, the source of this production in the blood, must be apparent. Of the general constitutional origin of cancer we fear there can be no doubt; but, in some few cases, it certainly does appear to commence as a purely local disease. However, it would be occupying too much time to consider this question at length, and, practically speaking, we may safely admit the constitutional origin and character of the affection.

The last part of the subject which occupies the attention of Dr. Carswell is the formation of carcinoma in the blood. Dr. Carswell adduces the following facts, as furnishing strong evidence that the formation of this substance takes place in the blood, whether it be found in this fluid alone, or in other parts of the body at the same time: 1st, the presence of this substance in the vessels which ramify in carcinomatous tumours, or in their immediate vicinity; 2d, in the vessels of a portion, or of the whole of an organ, to the former of which this substance is exclusively confined, and can be traced from the trunks into the branches and capillaries; 3d, in vessels having no direct communication with an organ affected with the same disease, as, for example, when it is confined to a small extent of the vena portæ; and, lastly, in blood which has been effused into the cellular tissue and on the surface of organs. The divisions of the vascular system in which the carcinomatous substance has been observed, are the venous and capillary.

We cannot admit that these arguments of Dr. Carswell are quite satisfactory, indeed we would venture to suggest that he is too hasty at generalization. Carcinomatous matter, says he, exists primitively in the blood, and from it is secreted. As a proof of this, he remarks that we find it in the blood. But it is singular that it is only found in the returned blood, in the venous system. The veins are rather organs of absorption than secretion, and Dr. Carswell might almost as well maintain, that, because in a case of compound fracture, we find pus in the veins of the limb, the pus originated in the blood, and was not conveyed from the part to the vein, or the product of mere inflammation of the tunics of the vein. Dr. Carswell must see that analogy is against him, and we fear the *formation* of morbid growths or morbid alterations of structure, is not yet to be explained so simply as Dr. Carswell would wish us to believe. Dr. C. remarks that the presence of an organized product in the blood can have no other origin than the blood, and that it cannot be introduced into this fluid by absorption or otherwise, are facts so obvious that he will not attempt further illustration of them. We may be very obtuse, but really these facts are not obvious to us.

How can a product be organized *in* the blood—do we find organized products in that fluid—as liver, or lung, or cellular tissue, or cerebral substance seen floating about in this fluid prior to its deposition? We apprehend not. Dr. Carswell cannot understand how an organized product can be introduced into the veins by absorption. But then this organized product is found in the absorbent vessels, and if in them why not in the veins? Surely the entry is as easy in the one case as in the other.

"The appearances which the carcinomatous formation presents in the blood are very various, and sometimes perfectly similar to those which mark its presence in the substance or on the surface of organs. In large veins, such as the vena portæ and its branches, the emulgent vein, &c. it may present the lardaceous, mammary, medullary, or hæmatoid characters, all in the same venous trunk. These varieties of the carcinomatous formation may be found mixed together in minute quantities, or isolated into masses so conspicuous that we can readily distinguish them from one another; sometimes they lie merely in contact with the internal parietes of the vein; at other times they are united to the latter by means of a thin layer of colourless fibrine; or minute blood-vessels pass from the one into the other, and are often very numerous and remarkably conspicuous in the cerebriform matter."

Dr. Carswell concludes by a criticism of the theories of Hodgkin, Baron, Abernethy, Andral, and Cruveilhier. It is indeed easy to point out the weak points in any of these theories, not excepting Dr. Carswell's, and as he has failed in such excellent company, he may reasonably console himself. Any theory on this subject must necessarily be a weak one, because it attempts to explain an ultimate fact. All such attempts in physic have failed and must fail.

Dr. Carswell intends to dedicate another fasciculus to the description of the physical, chemical, anatomical, and physiological characters of carcinoma. We cannot leave the present without congratulating the author and the professional public on the manner in which it is executed, though, if we might take the liberty, we would again suggest to Dr. Carswell to confine himself as much as possible to rigorous observation and induction, and to divest a work devoted to facts of theoretical considerations. He will do most justice to his subject and himself by bearing this in mind.

---

### XXI. RETENTION OF URINE PRODUCED BY IMPERFORATE HYMEN.*

This case is related by Mr. Coley, surgeon, of Bridgnorth. It is not uninteresting.

"March 25, 1832, I was requested to visit a young lady, aged 16, who resided at a considerable distance from this town. She had been ill three days and nights, with retention of urine; and her medical attendant had been under the necessity of relieving her by the introduction of the catheter, twice daily, during that period. The existence of so distressing a disease excited great apprehension; and my opinion was solicited respecting its nature and treatment. I found the cause of the ischury to consist of an imperforate hymen, which, by totally preventing the discharge of the menstrual fluid, had produced a mechanical obstruction in the urethra. The external orifice of the meatus urinarius was situated in a cul-de-sac, and the hymen was tense and slightly protruded. The bladder having been evacuated, I proceeded to examine the hypogastrium, where I discovered an obvious and considerable enlargement of the uterus of an oblong shape, extending nearly to the umbilicus. The lower part of the abdomen had been increasing in bulk during the last two years, and the breasts were fully developed; in short, she appeared to be in a state of pregnancy.

The patient being laid on her back, I pushed a double-edged scalpel through the hymen, which was very thick and tough; beginning at the upper part just

---

* Provincial Medical and Surgical Transactions, Vol. I.

below the meatus. Nearly four pints of tar-like fluid gushed out; after which I continued the incision down to the perineum. An aperture was thus made capable of admitting two fingers, into which a plug of lint was introduced."

Before the whole of the menstrual fluid was drawn off the young lady became hysterical, and so continued for four hours. The discharge ceased in a few days, a piece of sponge was introduced to keep asunder the sides of the vagina at the incisions, and the wound was healed by the 16th April. The hysterical fits continued for some days longer, when profuse menstruation occurred, soon after which the hysteria subsided.

Mr. Coley remarks that he has seen many cases of incomplete obstruction, in which there is a minute aperture at the upper portion of the hymen, through which part of the urine is forced out in drops or in a small stream, with great pain, resembling that produced by stone in the bladder. As the imperfection exists from the time of birth it is usually discovered when the child has attained the age of three or four years. In the cases which Mr. Coley has witnessed, a free incision effected a permanent cure. Sometimes the membrane is found double, sometimes of extraordinary density.

---

### XXII. Hysteria, Fatal.

In the twentieth fasciculus of his Principles of Obstetric Medicine, a work which we have often noticed, and which will form a valuable addition to obstetric works, an interesting instance of hysteria proving fatal is related by Dr. Davis. The case is taken from Villermay's work on Nervous Maladies, and as well authenticated facts of the sort are scarce, we are inclined to notice it.

A young woman, aged fifteen, presented the usual indications of ripened puberty. Her temperament was sanguineous; her figure was small, but well formed. She was active, alert, and cheerful; and she led the life of a domestic. When she was eight years old, she experienced an attack of convulsions, of which however she sustained no subsequent attacks until she arrived at the age of fourteen. She had then menstruated regularly, and in sufficient quantity, for about eight months; but pending her eighth period she became suppressed, in consequence of a fright. Nevertheless no great inconvenience was experienced at the time. On the next return of the function, however, the menstrual discharge presented itself only for a short time and in minute quantity, and was then immediately arrested. From that time forward she complained of feeling a general indisposition, accompanied by a sense of numbness in her lower extremities, thirst, and especially by expressions of concern for the interests of her situation as a servant. On the second day her case was aggravated towards evening by a painful sense of strangulation, such as might be produced by a tightly-drawn neckband or collar, and the respiration was consequently embarrassed. The hypogastric region became the seat of a marked fulness, and the extremities as well as the trunk of the body were repeatedly seized with convulsive movements. A strong spasmodic constriction of the pharynx prevented the patient from swallowing any liquid, although she was induced by much thirst to make strong and repeated efforts to accomplish that object. During this paroxysm she voided a great quantity of clear and limpid urine. On the third day, about mid-day, she was taken to the Hôtel-Dieu. Her sense of suffocation and her anxiety were then become very distressing; and she cried bitterly, complaining that she constantly felt as if being strangled. Her voice in the mean time was but little changed, and she moreover retained her reasoning faculties, and answered correctly to all the questions which were put to her. The convulsive movements of all the parts affected continued unmitigated. The abdomen was alternately elevated and depressed to a very remarkable degree. The poor patient constantly carried her hand to her neck, as if to remove the fatal collar. She constantly asked for something to drink, but her efforts to swallow were altogether ineffectual, the attempts in the mean time being extremely painful to her. Her difficulty of breathing was also undescribably distressing to her. She was every moment threatened with suffocation. Her pulse was contracted, hard, frequent, and very irregular. The movements of the heart could be felt to present similar characters, and they were perceptible to the touch as well as to view. The skin was of a reddish hue, and moist with perspiration. The unfortunate patient died in about six hours after her reception into the hospital, in the midst of a violent exacerbation of her disorder. She complained of having been strangled to the very last moment of her life.

Nothing was done for this young woman, and, with this exception, and with the exception also of the nature of the result, the case resembles very closely one related by Dr. Graves in an article which we have noticed in the present number of this Journal. We pray our readers to compare the cases. The body of this young woman was examined after death. The right cavities of the heart and pulmonary artery were "enormously charged" with black blood. The veins of the cerebrum and sinuses were also so charged. There was really nothing else of the slightest consequence, and the death of the poor girl, coupled with the fact that nothing was done for her in the Hôtel Dieu, is a cutting and melancholy satire on the médecine expectante.

---

XXIII. THE MEMORIAL OF THE PHYSICIANS PRACTISING IN LONDON,

Sheweth,

That the Royal College of Physicians in London was instituted by Charter, granted by Henry VIII., for the purpose of watching over the Interests of medical science, and promoting the respectability of the medical profession.

That by certain By-Laws framed by the College of Physicians, the Right of Admission into that Body is confined to persons who have been educated at the Universities of Oxford, Cambridge, and Dublin.

Your Memorialists are humbly of opinion, that the Right of Admission into this Corporation ought not to depend upon *religious tests*, but upon proof of good moral character, and adequate general and professional knowledge.

That your Memorialists, who have received their education in the University of Edinburgh, or in Foreign Schools celebrated for Medical Instruction, are subjected to the operation of By-Laws, framed in opposition to their interests, and professional advancement; that they are compelled to pay a considerable sum of money to a Corporation with which they have no connexion, over whose funds they have no control, and with whose laws and proceedings they are quite unacquainted.

Were it required, your Memorialists would have no difficulty in showing the many evils affecting the respectability of their Profession, and the advancement of Medical Science, which result from the narrow and exclusive system of monopoly exercised by the College of Physicians; the only conceivable consequence of which they feel persuaded is, to create jealousies and dissensions.

That your Memorialists have much pain in referring to the numerous conflicts which persons, similarly circumstanced as themselves, have had to maintain against the encroachments of a Corporation, whose obvious interest it is to narrow the field of public competition.

Your Memorialists beg to represent, that the numerous and artificial divisions in their profession, are the result of the exclusive privileges conferred on the different Corporations, the evils resulting from which they are most anxious to prove.

Your Memorialists, therefore, humbly pray, that such general inquiry may be instituted into the state of the Medical Profession, as may lead to the framing of Laws conducive to the interest of its Members, as well as the advantage of the Public.

We trust that the foregoing petition will be numerously signed, and sincerely do we hope that a full and fair enquiry into the state of the profession in this country may be the result.

